# Decreased expression of key tumour suppressor microRNAs is associated with lymph node metastases in triple negative breast cancer

**DOI:** 10.1186/1471-2407-14-51

**Published:** 2014-01-31

**Authors:** Kelly A Avery-Kiejda, Stephen G Braye, Andrea Mathe, John F Forbes, Rodney J Scott

**Affiliations:** 1 Centre for Information-Based Medicine, Hunter Medical Research Institute, New Lambton Heights, NSW 2305, Australia; 2 School of Biomedical Sciences and Pharmacy, Faculty of Health, University of Newcastle, Callaghan, NSW 2308, Australia; 3 Hunter Area Pathology Service, John Hunter Hospital, New Lambton Heights, NSW 2305, Australia; 4 Department of Surgical Oncology, Calvary Mater Newcastle Hospital, Australian New Zealand Breast Cancer Trials Group, Waratah, NSW 2298, Australia; 5 School of Medicine and Public Health, Faculty of Health, University of Newcastle, Callaghan, NSW 2308, Australia

**Keywords:** miRNA, Breast cancer, Triple negative, Metastases, Tumour suppressor

## Abstract

**Background:**

Breast cancer is the most common malignancy that develops in women, responsible for the highest cancer-associated death rates. Triple negative breast cancers represent an important subtype that have an aggressive clinical phenotype, are associated with a higher likelihood of metastasis and are not responsive to current targeted therapies. miRNAs have emerged as an attractive candidate for molecular biomarkers and treatment targets in breast cancer, but their role in the progression of triple negative breast cancer remains largely unexplored.

**Methods:**

This study has investigated miRNA expression profiles in 31 primary triple negative breast cancer cases and in 13 matched lymph node metastases compared with 23 matched normal breast tissues to determine miRNAs associated with the initiation of this disease subtype and those associated with its metastasis.

**Results:**

71 miRNAs were differentially expressed in triple negative breast cancer, the majority of which have previously been associated with breast cancer, including members of the miR-200 family and the miR-17-92 oncogenic cluster, suggesting that the majority of miRNAs involved in the initiation of triple negative breast cancer are not subtype specific. However, the repertoire of miRNAs expressed in lymph node negative and lymph node positive triple negative breast cancers were largely distinct from one another. In particular, miRNA profiles associated with lymph node negative disease tended to be up-regulated, while those associated with lymph node positive disease were down-regulated and largely overlapped with the profiles of their matched lymph node metastases. From this, 27 miRNAs were identified that are associated with metastatic capability in the triple negative breast cancer subtype.

**Conclusions:**

These results provide novel insight into the repertoire of miRNAs that contribute to the initiation of and progression to lymph node metastasis in triple negative breast cancer and have important implications for the treatment of this breast cancer subtype.

## Background

Breast cancer is the most common malignancy that develops in women worldwide, its incidence continues to rise and it is responsible for the highest cancer-associated death rates [1]. It is an extremely heterogeneous disease, made up of a number of different subtypes. Classification of breast cancer into subtypes can be partly attributed to the presence or absence of receptors for the hormones estrogen (ER), progesterone (PR) and human epidermal growth factor 2 (HER2). Triple negative breast cancer (TNBC) represents a particularly important clinical subtype, characterised by an absence of ER, PR and HER2 and which therefore lacks common targets used for anti-hormone therapies [2,3]. Although TNBCs comprise only a small percentage of all breast cancers diagnosed (10-24%), they have been recently the subject of intense investigation because of their aggressive clinical behaviour. Patients who are diagnosed with TNBC are of younger age, tend to develop tumours of larger size, and have an increased likelihood of distant metastasis and death within 5 years of diagnosis [2,3]. Thus, TNBCs represent a major clinical problem for which targeted therapies are currently not available.

microRNAs (miRNAs) are a class of small (~22 nucleotides) non-coding RNAs that control gene expression by targeting mRNAs and triggering either translational repression or RNA degradation [4]. miRNAs have emerged as an attractive candidate for molecular biomarkers and novel therapeutic targets in cancer because of their stability, ease of detection and their ability to act as endogenous antisense regulators of entire gene sets that regulate cancer growth [5].

Several studies have identified critical roles for miRNAs in breast cancer. Iorio and colleagues were the first to report significant deregulation in miRNA expression profiles in breast cancer when compared to normal breast tissue, where they showed that the expression of several miRNAs was associated with breast cancer subtypes and clinico-pathological features including hormone receptor status, clinical stage and proliferation index [6], suggesting that miRNA expression in breast cancer may have diagnostic and prognostic value. Since then, several miRNAs have been identified that have prognostic significance, including miR-210, miR-126, miR-21 and miR-205 [7-11]. However, the clinical cohorts used to generate these miRNA profiles have been highly heterogeneous, in regards to clinicopathological variables (e.g. grade, tumour size, subtype). Moreover, because these studies have concentrated on using miRNA profiles to better classify breast cancer subtypes, there are few studies that have profiled all miRNAs within a particular breast cancer subtype and even fewer that have used matched normal tissue as controls in these analyses; and so it is plausible that miRNAs important in disease initiation and progression that are subtype specific have been missed. TNBCs are distinct from ER-, PR- positive tumours at the molecular level and this classification has clinical implications [12]. Therefore, the identification of miRNAs that control TNBC initiation and progression could identify individuals that have more aggressive disease and may also help to identify subgroups of patients that are more responsive to particular treatments within these subtypes.

The dissemination of primary cancer cells to the lymphatic system represents one of the first signs of metastatic spread. In breast cancer, the number of positive lymph nodes (LN) is known to have an inverse linear correlation with prognosis and survival [13]. This is not the case with TNBC, where it has been shown that any LN involvement is associated with worse disease-free and overall survival [14]. Therefore, identification of miRNAs that are differentially expressed within LN metastases in this highly aggressive breast cancer subtype may serve as a better indicator of prognosis than miRNA profiles derived from primary breast cancers.

In this study, miRNA expression profiles of 31 primary TNBCs were examined and compared to the profiles of 23 matched normal breast tissues to reveal miRNAs that were differentially expressed in TNBC and that are potentially associated with its initiation. In addition, by comparing the miRNA expression profiles of lymph node positive primary tumours and matched LN metastases, we have identified a panel of miRNAs that are associated with metastatic capability in the TNBC subtype.

## Methods

### Study cohort and tissue sampling

Thirty-five formalin-fixed paraffin-embedded invasive ductal carcinomas (IDCs) were obtained from the archives of the Hunter Area Pathology Service, John Hunter Hospital, Newcastle, Australia. All patients were diagnosed with grade 3 IDC between the years of 2004–2009, and were negative for ER, PR and HER2 as assessed through routine diagnostic pathology (Additional file 1: Supplementary Methods and Figure S1). Areas of tissue representing histologically normal adjacent breast tissue (NAT, where available), IDC and breast cancer metastases (in LN) were identified and confirmed by a pathologist. Micrometastases (<2 mm) were not used in the analysis. None of the areas of selected NAT contained tumour tissue and all were enriched for terminal duct lobular units (>2 in biopsied area). Patient information is described in Table 1. A 1.5 mm punch biopsy was used to punch tissue cores from the paraffin blocks using haemotoxylin and eosin stained sections of the same sample for guidance. Tumour volume in the core biopsy was >70% of the total. This study complies with the Helsinki Declaration with ethical approval from the Hunter New England Human Research Ethics Committee (Approval number: 09/05/20/5.02). In accordance with the *National Statement on Ethical Conduct in Research Involving Humans*, a waiver of consent was granted for this study.

**Table 1:** Demographic data of triple negative breast cancer cases

**Patient information**	**Lymph node negative cases (16)**	**Lymph node positive cases (19)**
**Age (years)**		
Average (±SD)	58 ± 14	54 ± 14
Range	36–84	28–78
<50	4 (25%)	9 (47.3%)
50-69	8 (50%)	7 (36.8%)
>69	4 (25%)	3 (15.8%)
**Tumour size (mm)**		
Average (±SD)	32 ± 12	32 ± 21
Range	12–60	12–100
<20	2 (12.5%)	4 (21%)
20-39	10 (62.5%)	12 (63.2%)
>39	4 (25%)	3 (15.8%)
**No. of positive lymph nodes**		
1-3	0	13 (68.4%)
-Micrometastases (<2 mm) (No LN available)	0	4/13 (30.7%)
>3	0	6 (31.6%)
**Normal adjacent tissue**		
Yes	9 (56.3%)	15 (78.9%)
No	7 (43.8%)	4 (21.1%)

### Extraction of RNA

Total RNA was extracted using the miRNeasy FFPE kit (Qiagen, Doncaster, VIC, Australia). RNA was quantified using the Quant-it RiboGreen RNA Assay kit (Invitrogen, Mulgrave, VIC, Australia) and purity assessed by A_260/A280_ and A_260/230_ ratios (>1.8) using the Nanodrop. The RNA integrity of selected samples was analysed using the 2100 Bioanalyser and the RNA 6000 Nano kit (Agilent Technologies, Mulgrave, VIC, Australia).

### miRNA arrays

100 ng of total RNA was dephosphorylated and directly labelled with Cy3 using the miRNA Complete Labelling and Hyb Kit (Agilent Technologies). Labelled RNA was hybridised to Human miRNA microarrays (Sanger Release 14.0) according to the manufacturers’ instructions (Agilent Technologies) and scanned on an Agilent High-resolution C scanner.

### miRNA array analysis

These miRNA array results have been deposited in Gene Expression Omnibus (GEO) with Accession No. GSE38167. Data from 15,000 probe features representing 904 unique miRNAs was extracted using Agilent Feature Extraction software (v10.7.3.1) and converted to background subtracted signal intensities. The extracted data was imported into Genespring GX (Agilent Technologies) where it was log2 transformed and median normalised. Seven samples did not meet quality control measurements recommended by the manufacturer and were removed from the microarray analysis (1 IDC from a LN negative patient, 1 NAT and 3 IDCs from LN positive patients; and 2 LN metastases). Of all the probes interrogated in this analysis, those corresponding to 570 miRNA transcripts were present at a signal intensity threshold above background in at least one of the tissue samples. Unpaired *t*-tests were used to identify miRNAs with significantly altered expression (>2-fold, p < 0.05). To correct for false positive results, a Benjamini and Hochberg False Discovery Rate (FDR) of 5.0% was used for multiple testing. Supervised hierarchical cluster analysis was performed on miRNAs that were found to be significantly different (>2 fold, p < 0.05, FDR < 0.05). Similarity in the expression patterns between miRNAs was measured by Pearson’s correlation coefficient.

Biological targets of differentially expressed miRNAs were identified by searching for the presence of conserved 8mer and 7mer sites within genes that match the seed region of each miRNA. Non-conserved sites were also included in this analysis. This analysis was performed using sRNA Target Base (starBase, http://starbase.sysu.edu.cn/index.php) [15] which integrates data from 21 Ago or TNRC6 CLIP-Seq sequence data sets with the target prediction programs Target Scan, Pictar and miRanda. The number of genes identified by each of these programs and those in common between each of the programs is shown in Additional file 1: Figure S2. Only those target genes that were predicted by all three target prediction programs (560 genes) were used for further pathway analysis. PANTHER [16] was used to annotate the biological pathways that predicted miRNA target genes were involved in as previously described [17]. Pathways with a p-value <0.05 were considered to be significantly regulated by the miRNAs.

To determine the significance of differentially expressed miRNA families and clustered miRNAs; or to annotate the function of differentially expressed miRNAs, a freely available web-based resource, TAM (tool for annotations of miRNAs), was used [18].

### Semi-quantitative real-time PCR

Total RNA (5 ng) was reverse transcribed to generate cDNA using the Taqman MicroRNA Reverse Transcription Kit and Megaplex RT Human Primer Pools Set v3.0 (Applied Biosystems, Mulgrave, VIC, Australia) according to the manufacturers’ instructions. cDNA was amplified using the TaqMan PreAmp Master Mix with Megaplex Human PreAmp Primer Pools Set v3.0 (Applied Biosystems) according to the manufacturers’ instructions. Real-time PCR analysis was performed in triplicate on all samples (Table 1) using TaqMan Universal PCR mix No AmpErase UNG and TaqMan MicroRNA Assays (Applied Biosystems) according to the manufacturers’ instructions, with results quantified on a 7500 real-time PCR system (Applied Biosystems). The expression of the following miRNAs was analysed for array validation: hsa-let-7a (Assay ID: 377), hsa-let-7b (Assay ID: 2619), hsa-let-7c (Assay ID: 379), hsa-miR-100 (Assay ID: 437), hsa-miR-101 (Assay ID: 2253), hsa-miR-126* (Assay ID:451), hsa-miR-26a (Assay ID: 405), hsa-miR-26b (Assay ID: 407), hsa-miR-130a (Assay ID: 454), hsa-miR-29c (Assay ID: 587), hsa-miR-205 (Assay ID: 509), miR-210 (Assay ID: 512), RNU44 (Assay ID: 1094) and RNU49 (Assay ID: 1005). The relative miRNA expression was calculated by normalising the miRNA of interest to RNU44 (2^-ΔCt^). Relative expression of miRNAs was also calculated using a second normaliser, RNU49 (data not shown). The same relative expression patterns for the miRs analysed was observed when normalised with RNU49 (examples shown in Additional file 1: Figure S3). The relative expression of RNU44 and RNU49 was not significantly different between the subgroups analysed and therefore served as an appropriate normaliser for this analysis (Additional file 1: Figure S3). All pre-amplified multiplex miRNA assays were validated against uniplex miRNA assays to verify that the multiplex reaction did not affect miRNA quantitation (Additional file 1: Supplementary Methods, Table S1 and Figure S4). hsa-miR-126* and hsa-miR-205 were outside our range of acceptable PCR efficiencies and were not used for further validations.

### Statistical analysis

The normality of the data distribution was tested using a D’Agostino and Pearson Omnibus test. The values were found not to have been sampled from a Gaussian distribution and thus, non-parametric statistical tests were used to compare the real-time PCR data. A two-tailed Mann–Whitney U test was used to determine if there was a statistically significant difference in the expression of miRNAs between any two subgroups. The Kruskal-Wallis rank test followed by a Dunn’s Multiple Correction test was used to determine the statistical significance of miRNA expression between multiple (>2) subgroups. Analysis of the correlation between miRNA expression and clinical parameters was performed using Spearman’s correlation test. All analysis was performed using GraphPad Prism (version 5.04) software (GraphPad software Inc., La Jolla, CA, USA).

## Results

### miRNAs differentially expressed in TNBC

**Figure 1:**
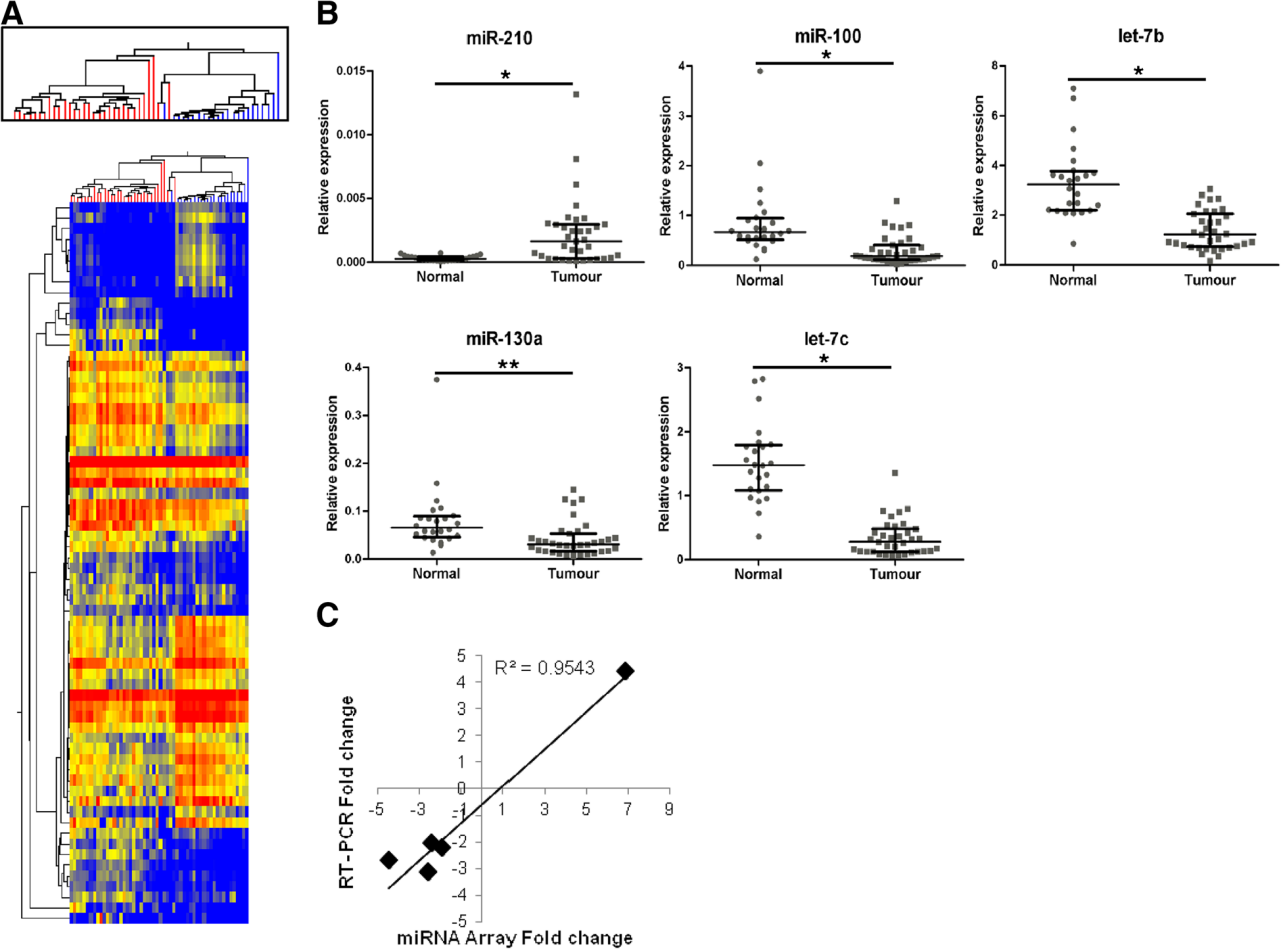
**miRNAs significantly different in triple negative breast cancer. A)** Supervised hierarchical cluster analysis was performed on 71 miRNAs significantly altered between tumour (n = 31, red branches, top) and normal adjacent tissue samples (n = 23, blue branches, top). Similarity in the expression patterns between miRNAs (branches shown on the left-hand panel) and between samples (branches shown on the top panel) was measured using Pearsons correlation. Distances between clustered branches represent the average distances between miRNAs and samples in the cluster. The height of each branch represents the degree of similarity within the cluster. miRNAs are coloured according to their expression level, where up-regulated expression is represented by red, down-regulated expression is represented by blue, and equal expression is represented by yellow. **B)** Relative quantification of miR-210, miR-100, miR-130a, let-7b and let-7c by real-time RT-PCR in normal (n = 24) and tumour samples (n = 35). Results are shown as a scatter plot of the relative normalised expression (target/RNU44) of the target miRNA (2^-ΔCt^). Values represent the median ± interquartile range. *p < 0.0001, **p = 0.0004. **C)** Correlation between real-time PCR and miRNA array results. Relative quantification of fold change (IDC v NAT) in miR-210, miR-100, miR-130a, let-7b and let-7c between normal and tumour samples by real-time RT-PCR (2^-ΔΔCt^, y-axis) or miRNA array (x-axis).

To identify miRNAs associated with TNBC, expression profiles were analysed in 35 grade 3 invasive ductal carcinomas (IDCs) compared to 24 matched normal adjacent tissue (NAT) specimens (Table 1). Seven samples did not meet quality control measurements recommended by the manufacturer and were removed from the microarray analysis (refer to Methods). Seventy-one of the miRNA transcripts were identified as being differentially expressed between IDC and NAT (Figure 1A). Supervised hierarchical clustering of these miRNAs clearly separated NAT from IDC, suggesting that the expression of these miRNAs can discriminate these two groups (Figure 1A). Five of the differentially expressed miRNAs (miR-210, miR-100, miR-130a, let-7b, let-7c) were verified by real-time PCR and were shown to be significantly different in expression between IDC and NAT in all cases (Figure 1B). In addition, the fold change in expression (IDC versus NAT) of the five miRNAs was highly concordant (R^2^ = 0.9543) between microarray and real-time PCR analysis, further confirming the validity of this approach (Figure 1C).

Of the 71 significantly altered miRNA transcripts, 39 showed increased expression and 32 showed decreased expression in IDC when compared to NAT (Additional file 1: Table S2). miRNAs are known to be located in genomic clusters [19]. Several of the miRNAs that were identified as being differentially expressed are clustered within the same genomic region, and four of these genomic clusters were significantly over-represented (p < 0.05): the miR-17-92 cluster (miR-17, miR-18a, miR19a, miR-19b, miR-20a), the miR-106b cluster (miR-25, miR-93, miR-106b), the miR-200a cluster (miR-200a, miR-200b, miR-429) and the miR-106a cluster (miR-18b, miR-19b, miR-20b, miR-363) (Additional file 1: Table S3). miRNAs are commonly grouped in families based on the similarity in their seed sequence. Three miRNA families were significantly (p < 0.05) over-represented: the miR-17 family (miR-17, miR-18a, miR-18b, miR-20a, miR-20b, miR-93, miR-106b), the miR-200 family (miR-200a, miR-200b, miR-200c, miR-141, miR-429) and the miR-130 family (miR-130a, miR-130b, miR-301a) (Additional file 1: Table S3). The majority of the differentially expressed miRNAs have previously been implicated in breast cancer including miR-200a, miR-200b, miR-200c, miR-21, miR-210, miR-205 and miR-10b [7,8,10,11,20,21]; and their regulation was concordant with previous studies. This suggests that the majority of the miRNAs identified in this study are relevant to the initiation of all breast cancers. In addition, we identified 5 miRNAs: miR-130a, miR-1280, miR-590-5p, miR-1308, miR-17*, which to the best of our knowledge, have not previously been implicated in breast cancer (Table 2). Of note, miR-130a has been shown to be associated with chemotherapy response in ovarian cancer and lung cancer cell lines, while miR-1280 has recently been demonstrated to inhibit invasion and metastasis by targeting ROCK1 when over-expressed in bladder cancer [22-24]. miR-590-5p has been reported to enhance (via the tumour suppressor PBRM1) or inhibit (via S100A10) cell growth and invasion depending on the cellular context, however, the function of miR-1308 and miR-17* has not been extensively studied [25,26].

**Table 2:** Unique miRNAs identified as being differentially expressed in triple negative breast cancer

**Systematic name**	**Fold regulation**	**p-value**
hsa-miR-130a	−2.21	0.0160
hsa-miR-1280	2.10	3.11E-04
hsa-miR-590-5p	2.15	0.0131
hsa-miR-1308	2.28	0.0035
hsa-miR-17*	3.03	3.11E-04

Fold change in expression of 5 miRNAs found to be significantly different in extracts from 31 TNBCs compared to extracts from 23 matched normal adjacent tissue specimens (>2-fold difference, p < 0.05 and a false discovery rate (FDR) = 5.0%).

### miRNAs differentially expressed in LN negative IDCs compared to LN positive IDCs

To determine miRNAs that were differentially expressed in patients with LN metastases, the primary breast cancers from 15 LN negative patients and 16 LN positive patients were compared. In this analysis, there were no miRNAs that were found to be significantly different. Additionally, the comparison of NAT from node negative and node positive women yielded no significant differences in miRNA profiles.

### miRNAs associated with LN metastasis

**Figure 2:**
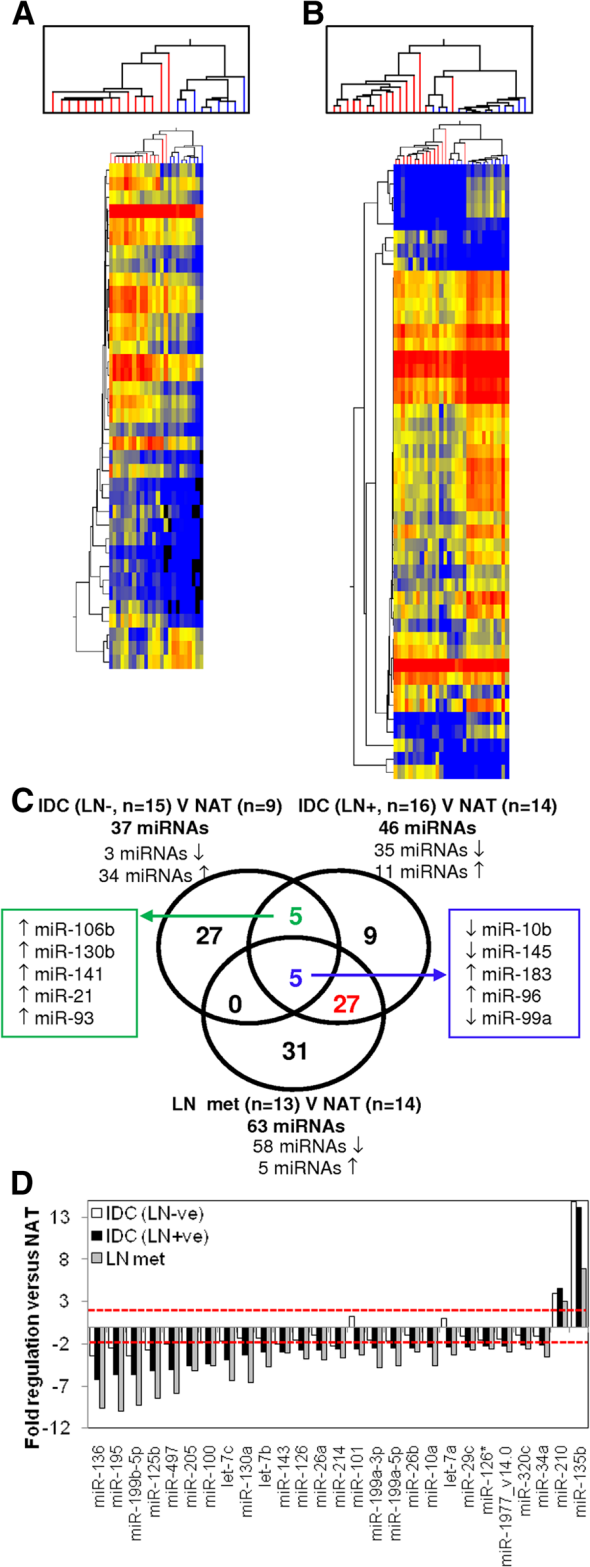
**miRNAs significantly different in triple negative breast cancer with lymph node metastases.** Supervised hierarchical cluster analysis was performed on miRNAs significantly altered between tumour (red branches, top) and normal adjacent tissue samples (blue branches, top) in **A)** lymph node negative and **B)** lymph node positive patients. Similarity in the expression patterns between miRNAs (branches shown on the left-hand panel) and between samples (branches shown on the top panel) was measured using Pearsons correlation. Distances between clustered branches represent the average distances between miRNAs and samples in the cluster. The height of each branch represents the degree of similarity within the cluster. miRNAs are coloured according to their expression level, where up-regulated expression is represented by red, down-regulated expression is represented by blue, and equal expression is represented by yellow. **C)** Venn diagram representing the overlap between miRNAs regulated in lymph node negative patients (37 miRNAs, left-hand circle), lymph node positive patients (46 miRNAs, right-hand circle) and in the lymph node metastases of lymph node positive patients (63 miRNAs, bottom circle). **D)** Histogram depicting the fold regulation of the 27 miRNAs (highlighted in red in **C**) in tumour compared to NAT in lymph node negative tumours (white), lymph node positive tumours (black) and lymph node metastases (grey). Two-fold regulation is depicted by the red line.

In contrast to the report of Cascione *et al*. we undertook an additional investigation examining the relationship between lymph node positivity and miRNA expression [27]. We determined the miRNA profiles of LN positive and LN negative IDCs compared to their matched NAT. From these analyses, 37 miRNAs were found to be altered in LN negative patients, while 46 miRNAs were found to be significantly different in LN positive patients (Figure 2, Additional file 1: Tables S4 and S5). Supervised hierarchical clustering of these miRNAs completely separated NAT from IDC into two distinct groups in LN negative patients (Figure 2A) and was able to distinguish the majority of LN positive IDCs (two IDCs were misclassified) from matched NAT (Figure 2B). Only 10 of the miRNAs that were identified as being differentially expressed in IDC versus NAT overlapped in LN negative and LN positive patients and the direction of their regulation (up, down) when compared to matched NAT, was concordant (Figure 2C, Additional file 1: Tables S4 and S5, miRNAs in bold). These included up-regulation of miR-21 and down-regulation of miR-10b; which have well known roles in breast cancer (Figure 2C; Additional file 1: Tables S4 and S5). Interestingly, the majority of miRNAs identified as being differentially expressed in LN negative patients were up-regulated (34/37 miRNAs), while the majority of differentially expressed miRNAs were down-regulated (35/46 miRNAs) in LN positive patients (Figure 2, Additional file 1: Tables S4 and S5). These results suggest that the repertoire of miRNAs whose expression is altered in the primary breast tumour when compared to the normal breast tissue is distinct in LN positive and negative patients.

Of note, the five unique miRNAs that were differentially regulated when all tumours were compared to all normal tissues (Table 2), were not commonly regulated when lymph node positive and negative cases were compared separately to matched normal tissue. Instead, the miRs that were positively regulated (miR-17*- 3.03, miR-590-5p- 2.15, miR-1280- 2.10) when all tumours were analysed became more highly up-regulated (miR-17*- 4.66, miR-590-5p- 3.13, miR-1280- 2.40) in the comparison of lymph node negative tumours versus matched normal tissue, but were not regulated in lymph node positive tumours. In contrast, while miR-130a was down-regulated in the comparison of all tumour tissues (−2.21), it became more strongly down-regulated in lymph node positive tumours (−3.32) when they were compared to matched normal tissues, and was not regulated in lymph node negative cases. This supports our data, that there are intrinsic differences between lymph node positive and negative TNBCs: miRs are mainly down-regulated in patients with lymph node metastases, and up-regulated in patients who do not have lymph node metastases and suggests that it is these patients (i.e. lymph node positive or negative tumours) that are driving the differential regulation (negative and positive respectively) when all patients are combined.

To determine miRNAs that were altered in LN metastases, the miRNA profile of LN metastases were compared to those of NAT (9 matched and 5 unmatched) from LN positive patients. From this analysis, 63 miRNAs were found to be differentially expressed (Additional file 1: Table S6). The majority of these miRNAs (58/63 miRNAs) were down-regulated and largely overlapped with the profile of their primary tumour (IDC, LN+; 32/63 miRNAs, Figure 2C). Many of the miRNAs overlap with those identified by Cascione *et al*., with 33% (21/63) of the differentially expressed miRNAs identified by our study also showing differential regulation in normal versus LN metastases comparisons in their study of TNBC [27]. No miRNAs were differentially expressed when LN positive primary IDCs and LN metastases were compared. This suggests that the profile of miRNAs differentially expressed in primary breast cancers are maintained in LN metastases from these tumours. This is also concordant with the results of Cascione *et al*. who found very few differentially expressed miRNAs in this comparison [27]. In contrast, only five of the 63 miRNAs that were differentially expressed in LN metastases were also differentially expressed in LN negative primary IDC’s (Figure 2C) when compared to NAT, suggesting that their miRNA profiles are largely distinct.

**Figure 3:**
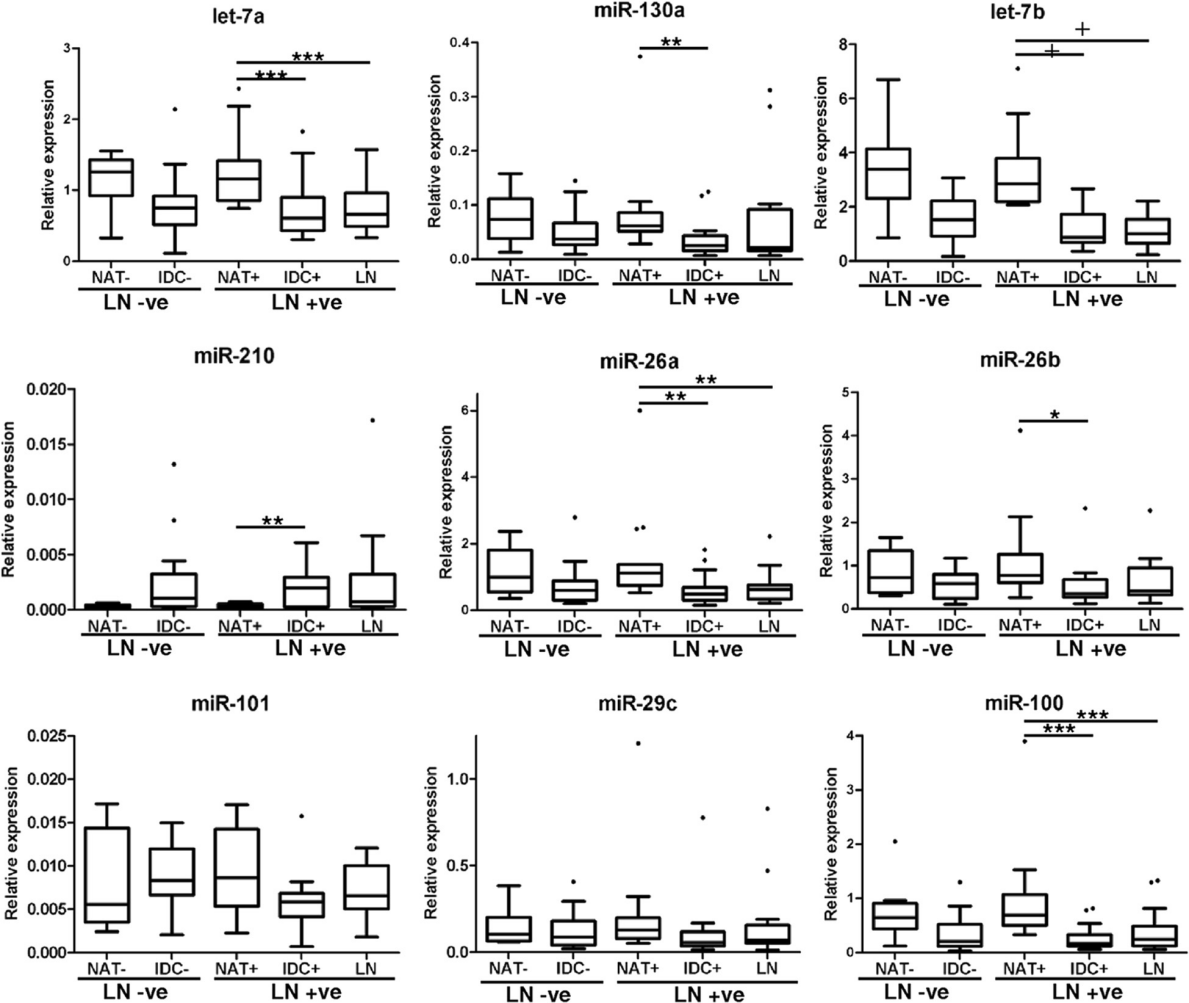
**Validation of selected miRNAs significantly different in triple negative breast cancer with lymph node metastases.** Relative quantification of let-7b, miR-130a, miR-101, miR-26a, miR-100, miR-210, miR-26b, miR-29c and let-7a by real-time RT-PCR in normal and tumour samples from lymph node negative and lymph node positive patients. Results are shown as a box plot of the relative normalised expression (target/RNU44) of the target miRNA (2^-ΔCt^). Boxes represent the median ± interquartile range. *p < 0.05, **p < 0.01, ***p < 0.0005 and ^+^p < 0.0001.

We reasoned that miRNAs that are differentially expressed in LN metastases and in the primary IDC of patients with LN metastases, that are not differentially expressed in patients who did not have LN metastases, may represent some of the earliest changes associated with metastatic progression. The overlap of the miRNA profiles is shown in Figure 2C, where 27 miRNAs were shown to meet these criteria. These concordantly regulated miRNAs include three members of the let-7 family (let-7a, -7b, -7c), two members of the miR-26 family (miR-26a, -26b) and three members of the miR-199 family (miR-199a-3p, -199a-5p, -199b-5p), suggesting that these miRNAs regulate similar target genes (Table 3). Furthermore, the list also contains miRNAs located within the same genomic cluster as one another, the let7a cluster (let-7a-3, -7b; and let-7a2, miR-100), the miR-195 cluster (miR-195, miR-497) and the miR-199a cluster (miR-199a, miR-214), suggesting they are co-ordinately regulated. With the exception of miR-210 and miR-135b, all miRNAs were significantly down regulated in the primary IDC and LN metastases when compared to matched NAT, but were not significantly different in primary IDCs from LN negative patients (Figure 2D and Table 3). These results were verified by real-time PCR for nine of the miRNAs (Figure 3), where the statistical significance in the difference in expression of these miRNAs was largely confirmed, with the exception of miR-101 and miR-29c, where the difference in expression of these miRNAs between normal and tumour samples in LN positive patients did not reach statistical significance.

**Table 3:** miRNAs differentially expressed in triple negative breast cancer patients with lymph node metastases

	**Lymph node positive cases**				**Lymph node negative cases**	
	**IDC v NAT**		**LN met v NAT**		**IDC v NAT**	
**Systematic name**	**Fold change**	**p-value**	**Fold change**	**p-value**	**Fold change**	**p-value**
hsa-let-7a	−2.36	0.00419	−3.33	0.00042	1.05	ns
hsa-let-7b	−2.99	0.00011	−4.67	0.00007	−1.30	ns
hsa-let-7c	−3.84	0.00006	−6.34	0.00001	−1.72	ns
hsa-miR-100	−4.37	0.00176	−4.57	0.00331	−1.84	ns
hsa-miR-101	−2.61	0.01750	−3.30	0.02278	1.22	ns
hsa-miR-10a	−2.37	0.02228	−4.64	0.00496	−1.78	ns
hsa-miR-125b	−5.18	0.00010	−8.41	0.00001	−2.69	ns
hsa-miR-126	−2.72	0.00451	−3.76	0.00451	−1.50	ns
hsa-miR-126*	−2.31	0.04409	−2.63	0.02725	−1.56	ns
hsa-miR-130a	−3.32	0.01446	−6.61	0.00430	−1.32	ns
hsa-miR-135b	14.14	0.00012	6.92	0.02725	14.87	ns
hsa-miR-136	−6.18	0.00011	−9.58	0.00223	−3.42	ns
hsa-miR-143	−2.99	0.01750	−3.04	0.03718	−2.02	ns
hsa-miR-195	−5.70	0.00006	−10.02	0.00029	−2.50	ns
hsa-miR-1977_v14.0	−2.28	0.00377	−2.91	0.00069	−1.49	ns
hsa-miR-199a-3p	−2.50	0.01750	−4.84	0.00451	−1.55	ns
hsa-miR-199a-5p	−2.46	0.04409	−4.64	0.00869	−1.66	ns
hsa-miR-199b-5p	−5.66	0.00011	−9.29	0.00021	−3.40	ns
hsa-miR-205	−4.65	0.02890	−5.18	0.01648	−1.82	ns
hsa-miR-210	4.56	0.00862	2.98	0.02389	4.01	ns
hsa-miR-214	−2.64	0.01360	−3.64	0.02023	−2.28	ns
hsa-miR-26a	−2.67	0.01428	−3.84	0.00247	−1.00	ns
hsa-miR-26b	−2.43	0.01681	−3.01	0.01308	−1.01	ns
hsa-miR-29c	−2.33	0.04343	−2.69	0.03676	−1.12	ns
hsa-miR-320c	−2.15	0.02461	−2.65	0.02023	−1.02	ns
hsa-miR-34a	−2.12	0.02087	−3.59	0.00825	−1.11	ns
hsa-miR-497	−5.11	0.00006	−7.91	0.00029	−2.06	ns

Fold change in expression of 27 miRNAs found to be significantly different in extracts from 16 TNBC cases (lymph node positive) and their corresponding lymph node metastases (13 cases) when compared to their matched normal adjacent tissue specimens (14 cases) (>2-fold difference, p < 0.05 and a false discovery rate (FDR) = 5.0%). Expression of these miRNAs in lymph node negative cases (IDC n = 15, NAT n = 9) is shown for comparison. ns = not significant.

### Biological functions and pathways regulated by the 27 miRNAs associated with LN metastasis

To annotate the function of the 27 miRNAs, we performed TAM analysis [18]. Through this analysis, the majority of miRNAs were found to function as tumour suppressor miRs (13/27 miRNAs) or were involved in human embryonic stem cell regulation (11/27 miRNAs) and these functions were significantly over-represented. A number of miRNAs in this list are also known to have functions in cell death and/or the cell cycle and these functions were also significantly over-represented (Additional file 1: Table S7).

We next used starBase and PANTHER to predict the genes and signalling pathways regulated by these miRNAs. Ten pathways were predicted to be significantly regulated by these miRNAs and many of these have already been reported to be dysregulated in TNBCs including the p53, EGFR1, Wnt and the TGFβ signalling pathways (Table 4).

**Table 4:** Predicted signalling pathways regulated by 27 differentially expressed miRNAs in TNBCs with LN metastases

**Pathway**	**Homo sapiens reference genome (No. of genes)**	**miRNA target genes (No. of genes)**	**Expected**	**Over/under represented (+/−)**	**p value**
Wnt signaling pathway	318	16	5.69	+	0.000248
TGF-beta signaling pathway	149	10	2.66	+	0.000423
Cell cycle	22	4	0.39	+	0.00072
p53 pathway	114	7	2.04	+	0.00487
p53 pathway by glucose deprivation	25	3	0.45	+	0.0106
p53 pathway feedback loops	52	4	0.93	+	0.0148
PI3 kinase pathway	117	6	2.09	+	0.0198
Heterotrimeric G-protein signaling pathway-Gi alpha and Gs alpha mediated pathway	164	7	2.93	+	0.0296
EGF receptor signaling pathway	130	6	2.32	+	0.0308
De novo pyrmidine ribonucleotides biosythesis	18	2	0.32	+	0.0418

### Correlation between miRNA expression and clinical parameters

To determine if the expression of miRNAs verified in this study by real-time PCR were independently related to age at diagnosis, tumour size or the percentage of positive LNs, we tested their association using Spearman’s Rank correlation (Table 5). None of the miRNAs tested were independently associated with age at diagnosis or tumour size. However, the expression of miR-101 was negatively correlated with the percentage of LNs positive while the negative correlation of let-7b and miR-29c with the percentage of LNs positive approached significance (Table 5).

**Table 5:** Correlation between miRNA expression levels and clinical variables in triple negative breast cancers

	**Age at diagnosis**		**Tumour size (mm)**		**% + ve lymph nodes**	
	**Spearman rank**	**p-value**	**Spearman rank**	**p-value**	**Spearman rank**	**p-value**
**let-7a**	0.04597	0.7932	0.07885	0.6525	−0.2904	0.0906
**let-7b**	−0.08997	0.6073	0.08432	0.6301	−0.3324	0.0511
**miR-100**	−0.09418	0.5905	0.171	0.3259	−0.1892	0.2764
**miR-101**	0.1756	0.313	0.17	0.3288	**−0.4355**	**0.0089**
miR-126*	0.0471	0.7882	0.08945	0.6093	−0.1581	0.3643
**miR-130a**	0.07147	0.6833	−0.1973	0.256	−0.2539	0.1411
**miR-210**	0.1029	0.5565	0.004349	0.9802	−0.09827	0.5744
**miR-26a**	0.05199	0.7668	0.08586	0.6238	−0.1696	0.3301
**miR-26b**	0.1511	0.3863	0.05724	0.744	−0.1778	0.3068
**miR-29c**	0.07231	0.6797	0.1254	0.4728	−0.3131	0.0671

Values represent Spearman’s rank correlation coefficient and significant correlations (p < 0.05) are shown in bold.

## Discussion

This study has investigated miRNA profiles in TNBC cases to determine miRNAs whose expression is associated with the initiation of this breast cancer subtype and those associated with its metastasis to the LN. We have identified a panel of 27 miRNAs that are associated with breast cancer progression in TNBC.

### miRNAs differentially expressed in TNBC

By comparing the miRNA profiles of primary breast tumours to matched normal adjacent breast tissue, 71 miRNAs were identified as being differentially expressed. The majority of the miRNAs identified in this analysis have previously been implicated in breast cancer, suggesting that these miRNAs have a general function in the tumourigenic process in all breast cancers and that they are not specific to TNBC. This is perhaps not surprising, given the weak separation of the TNBC subtype by hierarchical clustering in miRNA expression profiles when compared to other breast tumour subtypes [28]. The results of this study are highly concordant with that of Cascione *et al*., with 70% (50/71) of the differentially expressed miRNAs identified by our study also showing differential regulation in normal versus tumour comparisons in their study of TNBC [27]. Differentially expressed miRNAs included members of the miR-17-92 oncogenic cluster, the let-7 family and the miR-200 family which are known to be dysregulated in several solid tumours, including breast cancer [29,30]. Of particular note, the miR-17-92 cluster was recently shown to be up-regulated in TNBC and the basal subtype [10,31] while miR-145 and miR-199b-5p were strongly repressed [10] consistent with our results. miR-210 and miR-21, well known oncomiRs [7,11] were found to be over-expressed in our study. We also observed that miRNAs known to be *bona fide* regulators of ERα (miR-18a, miR-18b) were over-expressed [32]; while those that have been previously reported to be differentially expressed between ER-positive and -negative breast cancers (including let-7b, miR-200a, miR-21, miR-25, miR-106b) were significantly altered in this study [32].

The immunohistochemical and molecular profiles of TNBCs are similar to that of hereditary breast cancers that have mutations in the *BRCA1* gene [2]. In this regard, several miRNAs identified in this study are known to participate in the BRCA1 signalling axis including miR-146a, miR-155 and miR-335 [33-35]. Deregulation of these miRNAs in TNBC may contribute to altered BRCA1 signalling and could partly explain the similarities of these tumours with those in which BRCA1 function is lost.

### miRNAs involved in LN positive TNBC

A significant and important finding from this study is that the miRNA profiles of LN positive primary breast cancers were strikingly distinct from that of LN negative patients. In particular, there was an overall up-regulation of miRNAs in LN negative patients and a dampening of miRNA expression in LN positive patients, suggesting that oncogenic miRNAs are associated with the development of LN negative TNBC and in contrast, that deregulated expression of tumour suppressor miRs is involved in LN positive disease. Two enzymes, Drosha and Dicer, are pivotal in the processing of pri-miRNA into mature double stranded miRNA fragments [4]. Interestingly, in breast cancer, reduced expression of Dicer has been associated with shorter metastasis-free survival and with the TNBC subtype, where it is observed in 60-78% of patients [36-38]. The correlation of Dicer expression with LN metastases in TNBC patients was not examined in these studies [36,38]. It could be hypothesised that the overall down-regulation of miRNAs observed in LN positive TNBCs in the current study is a result of reduced Dicer expression, but this remains to be determined.

### miRNAs as markers for metastasis

In this study, we were not seeking to identify miRNAs that were differentially expressed in the transition of breast cancer progression i.e. primary breast cancer to metastasis or from ductal carcinoma in situ (DCIS) to IDC, as previous studies have done [10,27,28,39]. In contrast to other studies, our analysis yielded no discriminatory miRNAs when matched primary breast cancer and LN metastases were compared suggesting that they are remarkably similar in their miRNA profiles and supporting the validity of our approach. The premise of the current study was to determine altered miRNA expression patterns in primary breast cancers that were also present in LN metastases. We reasoned that deregulated expression of key miRNAs that promote a highly invasive phenotype would be an early event in breast cancer progression and that these changes would be present in both the primary and metastatic lesion. Using this approach, we identified a panel of 27 miRNAs that are associated with metastasis to the LN. Given that the majority of these miRNAs are down-regulated, their value as prognostic markers remains to be determined and is the subject of ongoing investigations. However, 17/27 (63%) miRNAs are either known to be tumour suppressor miRs and/or have been shown to play a role in human embryonic stem cell regulation (Additional file 1: Table S7), supporting that deregulated expression of this panel of miRNAs is likely to result in altered differentiation states and increased invasiveness. In this regard, let-7 family members, miR-34a and miR-205 are markedly reduced in breast tumour initiating cells and are involved in epithelial to mesenchymal transition [21,40,41]. In addition, several of the miRNAs in this list have already been shown to have prognostic value in breast cancer including miR-210, miR-126, miR-26a, miR-125b, miR-205 and miR-214 [7-10,42-44]. Furthermore, our results are in agreement with Cascione *et al.*, who found that both miR-125b and miR-497 were down-regulated and related to survival in TNBC [27].

Within our panel of metastasis-related miRNAs are a number of miRNAs whose functional role in breast cancer progression has not previously been described. These include miR-320c, miR-29c, miR-130a and miR-195 among others. There are no reports on miR-320c function to date and thus its possible role in promoting LN metastasis in TNBC remains unknown. miR-29c has previously been reported to be up-regulated in a small cohort of breast cancer cases [11], however, we found miR-29c to be down-regulated and associated with LN metastasis. In agreement with our study, miR-29c is down-regulated in chronic lymphocytic leukaemia and a range of solid tumours including colorectal cancer where it has been reported to decrease with cancer progression and is a predictor of survival and early recurrence [45,46]. miR-130a is down-regulated in a panel of ovarian cancer and lung cancer cell lines that are resistant to chemotherapies [23,24]. Moreover, it has recently been shown to be decreased in prostate cancer and its over-expression in prostate cancer cell lines causes the repression of key oncogenic pathways such as the MAPK pathway [47]. miR-195 was markedly down-regulated in our study. Its re-expression in breast cancer cell lines has recently been reported to reduce cellular proliferation and invasion, suggesting that it plays a key role in breast cancer progression [48].

A recent study by Buffa *et al.*, described three miRNAs (miR-342, miR-27b, miR-150) that were prognostic for relapse-free survival in TNBC [49]. In our hands, these results were not replicated for LN metastasis and moreover, these miRNAs were not differentially expressed when breast tumour was compared to normal tissue. In addition, miR-10b has been shown to be strongly expressed in metastatic breast cancer cells, where it regulates invasion and migration [50]. However, we observed miR-10b to be strongly down-regulated in lymph-node positive and negative primary breast cancers and in LN metastases, suggesting it does not contribute to breast cancer invasiveness, and this is in strong agreement with several recent studies [6,10,20].

We used starBASE to predict signalling pathways regulated by the 27 miRNAs associated with LN metastasis. Interestingly, the pathways identified by this analysis have been widely reported to be deregulated in TNBC. In particular Wnt/β-Catenin, EGFR1 and the TGFβ receptor signalling axis are all known to be highly expressed in a large proportion of cases within the TNBC subtype and have been proposed as alternative treatment targets for TNBC [2,3,51]. A number of chemotherapeutic agents which target these pathways are currently under investigation in patients with TNBC, in both the neoadjuvant and metastatic setting [52]. The findings from our study- decreased expression of key miRNAs which target these pathways- provides a biological mechanism for up-regulation of these pathways in this breast cancer subtype. Additionally, *TP53*, *PIK3CA* and *EGFR* are among the four most commonly mutated genes associated with aberrant expression of interacting genes in TNBC [53], exemplifying their importance in this subtype. The predicted regulation of these pathways by miRNAs, as found in our study, reveals another mechanism for disruption of these pathways in TNBC.

### Study design

A disadvantage of our study design is that samples were acquired by punch biopsy, rather than isolating individual normal breast and tumour epithelial cells by laser capture microdissection. We recognise that tissue heterogeneity and contamination by other non-neoplastic cell types including adipocytes, stroma and lymphocytes was unavoidable and that it may have contributed to the miRNA profiles generated. Our approach of selecting miRNAs that were only differentially expressed in primary tumours and matched LN metastases and that were not altered in LN negative primary tumours would have culled a large proportion of those miRNAs that are specifically altered in inflammatory responses. Moreover, those miRNAs that were differentially expressed simply because of differences in cell type composition between normal tissue (higher content of adipocytes and stroma) when compared to tumour tissue (higher content of epithelium) would have shown overlapping miRNA profiles when both LN negative and LN positive primary tumours were compared to matched normal tissues (10 miRNAs, Figure 2C) and were not included in our list of miRNAs related to metastasis (Table 3). Therefore, because of this careful process of elimination, we consider the miRNAs identified in this study (Table 3) are strongly representative of the invasive capacity of the primary tumour.

Given that the sample size used in this study is relatively small, conclusions cannot be drawn about the relationship of these miRNAs to prognosis or their specificity to the TNBC subtype. However, the tumours are homogenous with regards to size, hormone receptor status and histological grade. Furthermore, the size of this cohort (n = 35) is in line with several recent studies investigating the prognostic value of miRNAs in TNBC (Buffa *et al*., n = 37; Farazi *et al*., n = 48; Radojocic *et al*., n = 49) [20,28,49] and represents the only study to date that has analysed the full repertoire of miRNAs in lymph node positive and negative TNBC samples separately compared to matched normal adjacent tissue. The uniqueness of our study design was highly advantageous for delineating critical differences in miRNA profiles between LN positive and negative primary tumours, which would have been missed if: 1) matched normal adjacent tissue was not used and 2) only primary tumours from LN positive versus LN negative patients were compared to one another. In a recent study, Farazi and colleagues showed that there was no difference in miRNA profiles of primary breast tumours between TNBC patients who relapsed when compared to those who did not [28]. Indeed, we also found that no miRNAs were differentially expressed when LN negative versus positive primary tumours were directly compared using our statistical cut-offs. Further to this, in our study no miRNAs were found to be differentially expressed when normal tissue from LN positive patients was compared to that of LN negative patients. As such, the distinct miRNA profiles generated when LN negative and LN positive primary tumours were each separately compared to matched normal adjacent tissues are likely to reflect small, but critical changes in the regulation of miRNAs that are essential for the development of each of these respective phenotypes. Given that any LN involvement (regardless of number) is associated with worse disease-free and overall survival in TNBC [14], identification of molecular determinants such as miRNAs, that contribute to this highly invasive phenotype is urgently needed.

## Conclusions

This study has provided novel insight into the repertoire of miRNAs that contribute to the initiation of and progression to LN metastasis in TNBC. These miRNAs may serve as markers for metastasis or treatment targets in the future.

## Abbreviations

miRNA: Micro ribonucleic acid; ER: Estrogen receptor; PR: Progesterone receptor; HER2: Human epidermal growth factor receptor 2; TNBC: Triple negative breast cancer; mRNA: Messenger ribonucleic acid; LN: Lymph node; IDC: Invasive ductal carcinoma; NAT: Normal adjacent tissue; cDNA: Complementary deoxyribonucleic acid.

## Competing interests

The authors declare they have no competing interests.

## Authors’ contributions

KAK: study concept and design, carried out experiments, analysis and interpretation of data, drafting of the manuscript. SGB: patient collection, material support, manuscript revision. AM: analysis and interpretation of data. JFF: study design, obtained funding, critical revision of the manuscript for important intellectual content. RJS: study design, obtained funding, critical revision of the manuscript for important intellectual content. All authors read and approved the final manuscript.

## Pre-publication history

The pre-publication history for this paper can be accessed here:

http://www.biomedcentral.com/1471-2407/14/51/prepub

## Supplementary Material

Additional file 1 **Further characterisation of miRNAs in triple negative breast cancer.** Contains Supplementary methods, **Tables S1-S7** and **Figures S1-S4**. Click here for file

## References

[B1] Kamangar F Dores GM Anderson WF Patterns of cancer incidence, mortality, and prevalence across five continents: defining priorities to reduce cancer disparities in different geographic regions of the world J Clin Oncol 2006 24 14 2137 2150 10.1200/JCO.2005.05.2308 16682732

[B2] Podo F Buydens LM Degani H Hilhorst R Klipp E Gribbestad IS Van Huffel S van Laarhoven HW Luts J Monleon D Postma GJ Schneiderhan-Marra N Santoro F Wouters H Russnes HG Sorlie T Tagliabue E Borresen-Dale AL Triple-negative breast cancer: present challenges and new perspectives Mol Oncol 2010 4 3 209 229 10.1016/j.molonc.2010.04.006 20537966 PMC5527939

[B3] Carey L Winer E Viale G Cameron D Gianni L Triple-negative breast cancer: disease entity or title of convenience? Nat Rev Clin Oncol 2010 7 12 683 692 10.1038/nrclinonc.2010.154 20877296

[B4] Jackson RJ Standart N How do microRNAs regulate gene expression? Sci STKE 2007 2007 367 re1 17200520 10.1126/stke.3672007re1

[B5] Andorfer CA Necela BM Thompson EA Perez EA MicroRNA signatures: clinical biomarkers for the diagnosis and treatment of breast cancer Trends Mol Med 2011 17 6 313 319 10.1016/j.molmed.2011.01.006 21376668

[B6] Iorio MV Ferracin M Liu CG Veronese A Spizzo R Sabbioni S Magri E Pedriali M Fabbri M Campiglio M Menard S Palazzo JP Rosenberg A Musiani P Volinia S Nenci I Calin GA Querzoli P Negrini M Croce CM MicroRNA gene expression deregulation in human breast cancer Cancer Res 2005 65 16 7065 7070 10.1158/0008-5472.CAN-05-1783 16103053

[B7] Foekens JA Sieuwerts AM Smid M Look MP de Weerd V Boersma AW Klijn JG Wiemer EA Martens JW Four miRNAs associated with aggressiveness of lymph node-negative, estrogen receptor-positive human breast cancer Proc Natl Acad Sci USA 2008 105 35 13021 13026 10.1073/pnas.0803304105 18755890 PMC2529088

[B8] Le Quesne JL Jones J Warren J Dawson SJ Ali R Bardwell H Blows F Pharoah P Caldas C Biological and prognostic associations of miR-205 and let-7b in breast cancer revealed by in situ hybridisation analysis of micro-RNA expression in arrays of archival tumour tissue J Pathol 2012 227 3 306 314 10.1002/path.3983 22294324

[B9] Rothe F Ignatiadis M Chaboteaux C Haibe-Kains B Kheddoumi N Majjaj S Badran B Fayyad-Kazan H Desmedt C Harris AL Piccart M Sotiriou C Global microRNA expression profiling identifies MiR-210 associated with tumor proliferation, invasion and poor clinical outcome in breast cancer PLoS One 2011 6 6 e20980 10.1371/journal.pone.0020980 21738599 PMC3126805

[B10] Volinia S Galasso M Sana ME Wise TF Palatini J Huebner K Croce CM Breast cancer signatures for invasiveness and prognosis defined by deep sequencing of microRNA Proc Natl Acad Sci USA 2012 109 8 3024 3029 10.1073/pnas.1200010109 22315424 PMC3286983

[B11] Yan LX Huang XF Shao Q Huang MY Deng L Wu QL Zeng YX Shao JY MicroRNA miR-21 overexpression in human breast cancer is associated with advanced clinical stage, lymph node metastasis and patient poor prognosis RNA 2008 14 11 2348 2360 10.1261/rna.1034808 18812439 PMC2578865

[B12] Sorlie T Perou CM Tibshirani R Aas T Geisler S Johnsen H Hastie T Eisen MB van de Rijn M Jeffrey SS Thorsen T Quist H Matese JC Brown PO Botstein D Eystein Lonning P Borresen-Dale AL Gene expression patterns of breast carcinomas distinguish tumor subclasses with clinical implications Proc Nat Acad Sci USA 2001 98 19 10869 10874 10.1073/pnas.191367098 11553815 PMC58566

[B13] Carter CL Allen C Henson DE Relation of tumor size, lymph node status, and survival in 24,740 breast cancer cases Cancer 1989 63 1 181 187 10.1002/1097-0142(19890101)63:1<181::AID-CNCR2820630129>3.0.CO;2-H 2910416

[B14] Hernandez-Aya LF Chavez-Macgregor M Lei X Meric-Bernstam F Buchholz TA Hsu L Sahin AA Do KA Valero V Hortobagyi GN Gonzalez-Angulo AM Nodal status and clinical outcomes in a large cohort of patients with triple-negative breast cancer J Clin Oncol 2011 29 19 2628 2634 10.1200/JCO.2010.32.1877 21606433 PMC3139370

[B15] Yang JH Li JH Shao P Zhou H Chen YQ Qu LH starBase: a database for exploring microRNA-mRNA interaction maps from Argonaute CLIP-Seq and Degradome-Seq data Nucleic Acids Res 2011 39 Database issue D202 D209 21037263 10.1093/nar/gkq1056 PMC3013664

[B16] PANTHER Classification system http://www.pantherdb.org

[B17] Avery-Kiejda KA Bowden NA Croft AJ Scurr LL Kairupan CF Ashton KA Talseth-Palmer BA Rizos H Zhang XD Scott RJ Hersey P P53 in human melanoma fails to regulate target genes associated with apoptosis and the cell cycle and may contribute to proliferation BMC Cancer 2011 11 1 203 10.1186/1471-2407-11-203 21615965 PMC3120805

[B18] Lu M Shi B Wang J Cao Q Cui Q TAM: a method for enrichment and depletion analysis of a microRNA category in a list of microRNAs BMC Bioinformatics 2010 11 419 10.1186/1471-2105-11-419 20696049 PMC2924873

[B19] Altuvia Y Landgraf P Lithwick G Elefant N Pfeffer S Aravin A Brownstein MJ Tuschl T Margalit H Clustering and conservation patterns of human microRNAs Nucleic Acids Res 2005 33 8 2697 2706 10.1093/nar/gki567 15891114 PMC1110742

[B20] Radojicic J Zaravinos A Vrekoussis T Kafousi M Spandidos DA Stathopoulos EN MicroRNA expression analysis in triple-negative (ER, PR and Her2/neu) breast cancer Cell Cycle 2011 10 3 507 517 10.4161/cc.10.3.14754 21270527

[B21] Gregory PA Bert AG Paterson EL Barry SC Tsykin A Farshid G Vadas MA Khew-Goodall Y Goodall GJ The miR-200 family and miR-205 regulate epithelial to mesenchymal transition by targeting ZEB1 and SIP1 Nat Cell Biol 2008 10 5 593 601 10.1038/ncb1722 18376396

[B22] Majid S Dar AA Saini S Shahryari V Arora S Zaman MS Chang I Yamamura S Chiyomaru T Fukuhara S Tanaka Y Deng G Tabatabai ZL Dahiya R MicroRNA-1280 inhibits invasion and metastasis by targeting ROCK1 in bladder cancer PLoS One 2012 7 10 e46743 10.1371/journal.pone.0046743 23056431 PMC3464246

[B23] Acunzo M Visone R Romano G Veronese A Lovat F Palmieri D Bottoni A Garofalo M Gasparini P Condorelli G Chiariello M Croce CM miR-130a targets MET and induces TRAIL-sensitivity in NSCLC by downregulating miR-221 and 222 Oncogene 2012 31 5 634 642 21706050 10.1038/onc.2011.260 PMC3719419

[B24] Sorrentino A Liu CG Addario A Peschle C Scambia G Ferlini C Role of microRNAs in drug-resistant ovarian cancer cells Gynecol Oncol 2008 111 3 478 486 10.1016/j.ygyno.2008.08.017 18823650

[B25] Shan X Miao Y Fan R Qian H Chen P Liu H Yan X Li J Zhou F MiR-590-5P Inhibits Growth of HepG2 Cells via Decrease of S100A10 Expression and Inhibition of the Wnt Pathway Int J Mol Sci 2013 14 4 8556 8569 10.3390/ijms14048556 23598417 PMC3645761

[B26] Xiao X Tang C Xiao S Fu C Yu P Enhancement of proliferation and invasion by MicroRNA-590-5p via targeting PBRM1 in clear cell renal carcinoma cells Oncol Res 2013 20 11 537 544 24063284 10.3727/096504013X13775486749335

[B27] Cascione L Gasparini P Lovat F Carasi S Pulvirenti A Ferro A Alder H He G Vecchione A Croce CM Shapiro CL Huebner K Integrated microRNA and mRNA signatures associated with survival in triple negative breast cancer PLoS One 2013 8 2 e55910 10.1371/journal.pone.0055910 23405235 PMC3566108

[B28] Farazi TA Horlings HM Ten Hoeve JJ Mihailovic A Halfwerk H Morozov P Brown M Hafner M Reyal F van Kouwenhove M Kreike B Sie D Hovestadt V Wessels LF van de Vijver MJ Tuschl T MicroRNA sequence and expression analysis in breast tumors by deep sequencing Cancer Res 2011 71 13 4443 4453 10.1158/0008-5472.CAN-11-0608 21586611 PMC3129492

[B29] Blenkiron C Goldstein LD Thorne NP Spiteri I Chin SF Dunning MJ Barbosa-Morais NL Teschendorff AE Green AR Ellis IO Tavare S Caldas C Miska EA MicroRNA expression profiling of human breast cancer identifies new markers of tumor subtype Genome Biol 2007 8 10 R214 10.1186/gb-2007-8-10-r214 17922911 PMC2246288

[B30] Volinia S Calin GA Liu CG Ambs S Cimmino A Petrocca F Visone R Iorio M Roldo C Ferracin M Prueitt RL Yanaihara N Lanza G Scarpa A Vecchione A Negrini M Harris CC Croce CM A microRNA expression signature of human solid tumors defines cancer gene targets Proc Nat Acad Sci USA 2006 103 7 2257 2261 10.1073/pnas.0510565103 16461460 PMC1413718

[B31] Enerly E Steinfeld I Kleivi K Leivonen SK Aure MR Russnes HG Ronneberg JA Johnsen H Navon R Rodland E Makela R Naume B Perala M Kallioniemi O Kristensen VN Yakhini Z Borresen-Dale AL miRNA-mRNA integrated analysis reveals roles for miRNAs in primary breast tumors PLoS One 2011 6 2 e16915 10.1371/journal.pone.0016915 21364938 PMC3043070

[B32] Klinge CM miRNAs and estrogen action Trends Endocrinol Metab 2012 23 5 223 233 10.1016/j.tem.2012.03.002 22503553 PMC3348384

[B33] Garcia AI Buisson M Bertrand P Rimokh R Rouleau E Lopez BS Lidereau R Mikaelian I Mazoyer S Down-regulation of BRCA1 expression by miR-146a and miR-146b-5p in triple negative sporadic breast cancers EMBO Mol Med 2011 3 5 279 290 10.1002/emmm.201100136 21472990 PMC3377076

[B34] Chang S Wang RH Akagi K Kim KA Martin BK Cavallone L Haines DC Basik M Mai P Poggi E Isaacs C Looi LM Mun KS Greene MH Byers SW Teo SH Deng CX Sharan SK Tumor suppressor BRCA1 epigenetically controls oncogenic microRNA-155 Nat Med 2011 17 10 1275 1282 10.1038/nm.2459 21946536 PMC3501198

[B35] Heyn H Engelmann M Schreek S Ahrens P Lehmann U Kreipe H Schlegelberger B Beger C MicroRNA miR-335 is crucial for the BRCA1 regulatory cascade in breast cancer development Int J Cancer 2011 129 12 2797 2806 10.1002/ijc.25962 21618216

[B36] Dedes KJ Natrajan R Lambros MB Geyer FC Lopez-Garcia MA Savage K Jones RL Reis-Filho JS Down-regulation of the miRNA master regulators Drosha and Dicer is associated with specific subgroups of breast cancer Eur J Cancer 2011 47 1 138 150 10.1016/j.ejca.2010.08.007 20832293

[B37] Grelier G Voirin N Ay AS Cox DG Chabaud S Treilleux I Leon-Goddard S Rimokh R Mikaelian I Venoux C Puisieux A Lasset C Moyret-Lalle C Prognostic value of Dicer expression in human breast cancers and association with the mesenchymal phenotype Br J Cancer 2009 101 4 673 683 10.1038/sj.bjc.6605193 19672267 PMC2736830

[B38] Passon N Gerometta A Puppin C Lavarone E Puglisi F Tell G Di Loreto C Damante G Expression of Dicer and Drosha in triple-negative breast cancer J Clin Pathol 2012 65 4 320 326 10.1136/jclinpath-2011-200496 22259182

[B39] Baffa R Fassan M Volinia S O'Hara B Liu CG Palazzo JP Gardiman M Rugge M Gomella LG Croce CM Rosenberg A MicroRNA expression profiling of human metastatic cancers identifies cancer gene targets J Pathol 2009 219 2 214 221 10.1002/path.2586 19593777

[B40] Kim NH Kim HS Li XY Lee I Choi HS Kang SE Cha SY Ryu JK Yoon D Fearon ER Rowe RG Lee S Maher CA Weiss SJ Yook JI A p53/miRNA-34 axis regulates Snail1-dependent cancer cell epithelial-mesenchymal transition J Cell Biol 2011 195 3 417 433 10.1083/jcb.201103097 22024162 PMC3206336

[B41] Yu F Yao H Zhu P Zhang X Pan Q Gong C Huang Y Hu X Su F Lieberman J Song E let-7 regulates self renewal and tumorigenicity of breast cancer cells Cell 2007 131 6 1109 1123 10.1016/j.cell.2007.10.054 18083101

[B42] Zhang Y Yan LX Wu QN Du ZM Chen J Liao DZ Huang MY Hou JH Wu QL Zeng MS Huang WL Zeng YX Shao JY miR-125b is methylated and functions as a tumor suppressor by regulating the ETS1 proto-oncogene in human invasive breast cancer Cancer Res 2011 71 10 3552 3562 10.1158/0008-5472.CAN-10-2435 21444677

[B43] Jansen MP Reijm EA Sieuwerts AM Ruigrok-Ritstier K Look MP Rodriguez-Gonzalez FG Heine AA Martens JW Sleijfer S Foekens JA Berns EM High miR-26a and low CDC2 levels associate with decreased EZH2 expression and with favorable outcome on tamoxifen in metastatic breast cancer Breast Cancer Res Treat 2012 133 3 937 947 10.1007/s10549-011-1877-4 22094936 PMC3387494

[B44] Schwarzenbach H Milde-Langosch K Steinbach B Muller V Pantel K Diagnostic potential of PTEN-targeting miR-214 in the blood of breast cancer patients Breast Cancer Res Treat 2012 134 3 933 941 10.1007/s10549-012-1988-6 22350790

[B45] Kuo TY Hsi E Yang IP Tsai PC Wang JY Juo SH Computational analysis of mRNA expression profiles identifies microRNA-29a/c as predictor of colorectal cancer early recurrence PLoS One 2012 7 2 e31587 10.1371/journal.pone.0031587 22348113 PMC3278467

[B46] Stamatopoulos B Meuleman N Haibe-Kains B Saussoy P Van Den Neste E Michaux L Heimann P Martiat P Bron D Lagneaux L microRNA-29c and microRNA-223 down-regulation has in vivo significance in chronic lymphocytic leukemia and improves disease risk stratification Blood 2009 113 21 5237 5245 10.1182/blood-2008-11-189407 19144983

[B47] Boll K Reiche K Kasack K Morbt N Kretzschmar AK Tomm JM Verhaegh G Schalken J von Bergen M Horn F Hackermuller J MiR-130a, miR-203 and miR-205 jointly repress key oncogenic pathways and are downregulated in prostate carcinoma Oncogene 2013 32 3 277 285 10.1038/onc.2012.55 22391564

[B48] Li D Zhao Y Liu C Chen X Qi Y Jiang Y Zou C Zhang X Liu S Wang X Zhao D Sun Q Zeng Z Dress A Lin MC Kung HF Rui H Liu LZ Mao F Jiang BH Lai L Analysis of MiR-195 and MiR-497 expression, regulation and role in breast cancer Clin Cancer Res 2011 17 7 1722 1730 10.1158/1078-0432.CCR-10-1800 21350001

[B49] Buffa FM Camps C Winchester L Snell CE Gee HE Sheldon H Taylor M Harris AL Ragoussis J microRNA-associated progression pathways and potential therapeutic targets identified by integrated mRNA and microRNA expression profiling in breast cancer Cancer Res 2011 71 17 5635 5645 10.1158/0008-5472.CAN-11-0489 21737487

[B50] Ma L Teruya-Feldstein J Weinberg RA Tumour invasion and metastasis initiated by microRNA-10b in breast cancer Nature 2007 449 7163 682 688 10.1038/nature06174 17898713

[B51] King TD Suto MJ Li Y The Wnt/beta-catenin signaling pathway: a potential therapeutic target in the treatment of triple negative breast cancer J Cell Biochem 2012 113 1 13 18 10.1002/jcb.23350 21898546 PMC10924801

[B52] Rakha EA Chan S Metastatic triple-negative breast cancer Clin Oncol 2011 23 9 587 600 10.1016/j.clon.2011.03.013 21524569

[B53] Shah SP Roth A Goya R Oloumi A Ha G Zhao Y Turashvili G Ding J Tse K Haffari G Bashashati A Prentice LM Khattra J Burleigh A Yap D Bernard V McPherson A Shumansky K Crisan A Giuliany R Heravi-Moussavi A Rosner J Lai D Birol I Varhol R Tam A Dhalla N Zeng T Ma K Chan SK The clonal and mutational evolution spectrum of primary triple-negative breast cancers Nature 2012 486 7403 395 399 22495314 10.1038/nature10933 PMC3863681

