# Supplementary material for: Decreased expression of key tumour suppressor microRNAs is associated with lymph node metastases in triple negative breast cancer
Source: BMC Cancer. 2014 Jan 31;14:51. doi: 10.1186/1471-2407-14-51 (PMC3912930; doi:10.1186/1471-2407-14-51)

Supplementary Data

Supplementary Methods

Immunohistochemistry

To assess hormone receptor levels in formalin-fixed paraffin-embedded tumour tissue specimens, sections were deparaffinised and stained using the Benchmark ULTRA IHC/ISH automated slide staining module (Benchmark ULTRA, Ventana Medical Systems Inc., Tucson, AZ, USA) according to the manufacturers' instructions. The U *ultra*View DAB procedure was performed. This included: treatment with ULTRA Cell Conditioner #1 at 95°C for 8 mins to retrieve antigenicity; followed by incubation at 36°C with the specific primary rabbit monoclonal antibody (Ventana) for ER (24 mins, CONFIRM™ anti-ER (SP1), 1 μg/mL in 0.05 M Tris-HCl), PR (12 mins, CONFIRM™ anti-PR (1E2), 1 μg/mL in 0.05 M Tris-HCl) or HER2 (40 mins, CONFIRM™ anti-HER2 (4B5), 6 μg/mL in 0.05 M Tris-HCl) and development with the *ultra*View Universal DAB Detection Kit (Ventana). Positive and negative tissue controls were included in all staining procedures. Results were reviewed on a light microscope by a pathologist as part of routine clinical diagnosis. ER and PR expression was recorded as the percentage of stained tumour cells within an entire section. Cases were classified as positive for expression of the receptors (≥1% staining cells) or negative (<1%), irrespective of the staining intensity. HER2 expression was defined by the intensity level of the staining where it was scored 0 (no staining or staining in <10% of tumour cells), 1+ (faint staining in >10% of tumour cells or partial membrane staining), 2+ (weak to moderate staining in >10% of tumour cells) or 3+ (strong, complete membrane staining in >10% of tumour cells), as outlined by Boström *et al* (BMC Cancer 2011; 11:348).

Silver in situ hybridisation

To determine if the *HER2* gene was amplified in formalin-fixed paraffin-embedded tumour tissue specimens, sections were deparaffinised and stained using the Benchmark ULTRA IHC/ISH automated slide staining module (Benchmark ULTRA, Ventana Medical Systems Inc., Tucson, AZ, USA) according to the manufacturers' instructions. The *ultra*View HER2 SISH procedure was performed. This included: hybridisation of serial tissue sections with either the Dinitro-Phenol (DNP) labelled INFORM *HER2*

DNA probe (10 µg/mL, Ventana) for 6 hours at 52°C; followed by visualisation of the DNP-labelled probe using the *ultra*View SISH Detection Kit (Ventana) according to the manufacturers' instructions. All reagents have been optimally formulated for use on the Benchmark ULTRA automated slide stainer (Ventana). Results were reviewed on a light microscope by a pathologist as part of routine clinical diagnosis.The internal control for the SISH staining was verified with 2 copy signals identified in normal stromal or lymphoid cells. The number of *HER2* signals was then counted in thirty tumour nuclei, and scored as the average number of signals per nuclei; where a score of 1-4 copies/nuclei was classed as non-amplified (either diploid or polysomic for Chromosome 17 depending upon count numbers below or above 2.5 respectively), 4-6 copies/nuclei was classed as equivocal and >6 copies/nuclei was classed as amplified. In cases with 4-6 copies/nuclei, an additional 30 nuclei were examined and counted, bringing the average copy number calculation across a total of 60 cells. If the average copy number still then lay between 4 and 6, additional staining for Chromosome 17 (INFORM Chromosome 17 DNA probe; 10 µg/mL, Ventana) was undertaken to determine if polysomy was present. In these cases, *HER2* gene status was classed as negative (*HER2*:Chr17<1.8), equivocal (*HER2*:Chr17=1.8-2.2) or positive (*HER2*:Chr17>2.2) for *HER2* gene amplification.

Validation of miRNA multiplexed pre-amplified real-time PCR assays

To verify that the multiplex pre-amplification procedure did not alter the quantitation of the miRNAs used in the real time PCR assays, all pre-amplified multiplex assays were validated against the uniplex miRNA assays in triplicate. To do this, a standard curve was generated using 10-fold serially diluted pre-amplified or non-amplified reverse transcribed total RNA from T-47D breast cancer cells. A standard curve was prepared by plotting the Log_{10} template amount versus Ct. PCR efficiencies were calculated using the slope of the standard curve and the following equation: PCR efficiency (%) = $(10^{-1/slope}-1) \times 100$. A PCR efficiency of 75-125% for an individual miRNA assay was considered acceptable (as per Sieuwerts *et al*, Clin Cancer Res 2011;17: 3600-18). hsa-miR-126* and hsa-miR-205 were outside our range of acceptable PCR efficiencies and were not used for further validations. PCR linearity was determined using R^2. Results are shown in Supplementary Table S1 and Supplementary Figure S3.

Supplementary Table S1: Validation of miRNA real-time PCR assay efficiency before and after multiplexed pre-amplification procedure.

miRNA assay	Median Ct in uniplex reaction without pre-amplification	Median Ct in multiplex reaction with pre-amplification	Ct gain due to multiplex pre-amplification	PCR efficiency (%) in uniplex reaction without pre-amplification	PCR efficiency (%) in multiplex reaction with pre-amplification	Linearity (R^2) in uniplex reaction without pre-amplification	Linearity (R^2) in multiplex reaction with pre-amplification
**let-7a**	23.80	20.45	3.35	92.37	78.27	0.9708	0.9844
**let-7b**	25.76	22.70	3.06	113.20	80.41	0.9777	0.9796
**let-7c**	29.19	24.96	4.23	110.00	77.11	0.9350	0.9724
**mir-100**	26.05	22.57	3.48	102.92	85.53	0.9778	0.9873
**mir-101**	29.38	25.50	3.87	112.82	96.11	0.9714	0.9309
**mir-26a**	26.29	18.65	7.64	82.73	99.83	0.9636	0.9973
**mir-26b**	27.41	20.67	6.75	90.36	82.84	0.9240	0.9982
**miR-130a**	27.13	20.12	7.01	97.13	85.43	0.9366	0.9894
**miR-29c**	26.40	20.44	5.96	87.62	97.38	0.9719	0.9873
**miR-205**	26.25	20.08	6.17	117.58	418.43	0.9877	0.8377
**RNU44**	25.61	16.92	8.69	101.04	124.39	0.9891	0.9928
**mir-126***	33.73	27.23	6.50	96.09	43.76	0.8255	0.9603
**miR-210**	25.83	20.73	5.10	87.04	75.10	0.9941	0.9668

Supplementary Table S2: miRNAs differentially expressed in triple negative breast cancer. Fold change in expression of 71 miRNAs found to be significantly different in extracts from 31 TNBCs compared to extracts from 23 matched normal adjacent tissue specimens (>2-fold difference, $p<0.05$ and a false discovery rate (FDR) = 5.0%). miRNAs with no previous association with breast cancer are shown in bold.

Up-regulated miRNAs			Down-regulated miRNAs		
**Systematic name**	**Fold regulation**	**p-value**	**Systematic name**	**Fold regulation**	**p-value**
hsa-miR-151-3p	2.02	0.0136	hsa-miR-451	-5.51	1.42E-04
hsa-miR-185	2.03	0.0124	hsa-miR-145	-5.24	3.16E-06
hsa-miR-660	2.05	0.0160	hsa-miR-136	-4.80	1.18E-05
**hsa-miR-1280**	**2.10**	**3.11E-04**	hsa-miR-10b	-4.72	1.62E-07
**hsa-miR-590-5p**	**2.15**	**0.0131**	hsa-miR-199b-5p	-4.65	3.21E-06
hsa-miR-1290	2.15	0.0249	hsa-miR-99a	-4.44	9.37E-07
hsa-miR-200b	2.19	0.0054	hsa-miR-195	-3.95	3.21E-06
hsa-miR-425	2.23	0.0016	hsa-miR-125b	-3.94	2.14E-06
hsa-miR-19b	2.23	0.0090	hsa-miR-497	-3.43	4.30E-05
**hsa-miR-1308**	**2.28**	**0.0035**	hsa-miR-376a	-3.38	0.0098
hsa-miR-15b	2.29	7.56E-04	hsa-miR-376c	-3.34	0.0125
hsa-miR-663	2.35	0.0125	hsa-miR-127-3p	-3.25	0.0019
hsa-miR-200c	2.40	2.32E-04	hsa-miR-205	-3.14	0.0096
hsa-miR-20a	2.48	0.0017	hsa-miR-100	-3.01	0.0017
hsa-miR-200a	2.51	0.0096	hsa-miR-335	-2.77	0.0128
hsa-miR-146a	2.52	0.0099	hsa-let-7c	-2.71	4.50E-06
hsa-miR-25	2.60	3.11E-04	hsa-miR-542-3p	-2.69	0.0021
hsa-miR-20b	2.76	0.0030	hsa-miR-377	-2.69	0.0369
hsa-miR-155	2.81	0.0016	hsa-miR-450a	-2.62	0.0249
hsa-miR-301a	2.84	0.0016	hsa-miR-143	-2.61	0.0032
hsa-miR-429	2.91	0.0017	hsa-miR-214	-2.53	0.0012
hsa-miR-142-3p	2.98	0.0017	hsa-miR-299-5p	-2.53	0.0240
hsa-miR-7	3.00	0.0304	hsa-miR-486-5p	-2.39	0.0283
**hsa-miR-17***	**3.03**	**3.11E-04**	hsa-miR-132	-2.38	0.0284
hsa-miR-141	3.30	4.56E-05	hsa-miR-542-5p	-2.35	0.0049
hsa-miR-106b	3.45	2.95E-05	hsa-miR-424	-2.23	0.0132
hsa-miR-19a	3.50	1.55E-04	**hsa-miR-130a**	**-2.21**	**0.0160**
hsa-miR-130b	3.54	2.31E-06	hsa-miR-10a	-2.14	0.0028
hsa-miR-93	3.59	7.43E-06	hsa-miR-199a-5p	-2.14	0.0189
hsa-miR-17	3.63	3.61E-05	hsa-miR-126	-2.11	0.0026
hsa-miR-363	3.66	0.0284	hsa-miR-199a-3p	-2.08	0.0098
hsa-miR-21	3.74	7.94E-07	hsa-let-7b	-2.06	0.0011
hsa-miR-203	3.87	0.0136			
hsa-miR-210	4.41	0.0012			
hsa-miR-18b	4.64	0.0026			
hsa-miR-18a	6.24	0.0455			
hsa-miR-96	6.29	1.62E-07			
hsa-miR-183	7.66	1.62E-07			
hsa-miR-135b	11.57	5.73E-04			

Supplementary Table S3: miRNA families and clustered miRNAs differentially expressed in triple negative breast cancer.

Systematic name	No. of miRNAs	Fold over-representation	FDR corrected p-value
Clustered miRNAs			
hsa-mir-17-92 cluster	5	6.83	1.39E-03
hsa-mir-200a cluster	3	8.20	0.0118
hsa-mir-106b cluster	3	8.20	0.0119
hsa-mir-106a cluster	4	5.46	0.0167
miRNA families			
mir-17 family	7	7.17	5.11E-05
mir-200/miR-8 family	5	8.20	3.64E-04
mir-130 family	3	6.15	0.0382

Supplementary Table S4: miRNAs differentially expressed in lymph node negative triple negative breast cancer. Fold change in expression of 37 miRNAs found to be significantly different in extracts from 15 lymph node negative TNBCs compared to 9 matched normal adjacent tissue specimens (>2-fold difference, $p<0.05$ and a false discovery rate (FDR) = 5.0%). miRNAs differentially expressed in lymph node positive patients are shown in bold.

Systematic name	Fold change	p-value
**hsa-miR-106b**	**5.40**	**0.00348**
hsa-miR-107	2.51	0.04619
**hsa-miR-10b**	**-3.60**	**0.01790**
hsa-miR-1280	2.40	0.02725
**hsa-miR-130b**	**4.51**	**0.00223**
**hsa-miR-141**	**5.78**	**0.00372**
hsa-miR-142-3p	4.78	0.00850
**hsa-miR-145**	**-3.77**	**0.02881**
hsa-miR-146a	4.76	0.00719
hsa-miR-155	3.60	0.02881
hsa-miR-15b	3.23	0.00531
hsa-miR-17	6.09	0.00204
hsa-miR-17*	4.66	0.00820
hsa-miR-181a	2.30	0.02881
hsa-miR-181b	2.54	0.02881
**hsa-miR-183**	**11.46**	**0.00062**
hsa-miR-185	3.05	0.03114
hsa-miR-1979	2.57	0.03832
hsa-miR-19a	5.49	0.00369
hsa-miR-19b	3.77	0.00531
hsa-miR-200a	3.76	0.04956
hsa-miR-200b	3.02	0.04619
hsa-miR-200c	3.77	0.00531
hsa-miR-20a	4.20	0.00348
hsa-miR-20b	5.11	0.00223
**hsa-miR-21**	**4.86**	**0.00204**
hsa-miR-25	4.01	0.00495
hsa-miR-301a	3.99	0.02186
hsa-miR-425	2.76	0.04331
hsa-miR-454	2.83	0.00695
hsa-miR-590-5p	3.13	0.01780
hsa-miR-660	2.78	0.04619
hsa-miR-92a	2.73	0.01755
**hsa-miR-93**	**5.33**	**0.00348**
**hsa-miR-96**	**9.52**	**0.00027**
hsa-miR-98	2.67	0.02576
**hsa-miR-99a**	**-3.19**	**0.03013**

Supplementary Table S5: miRNAs differentially expressed in lymph node positive triple negative breast cancer. Fold change in expression of 46 miRNAs found to be significantly different in extracts from 16 lymph node positive triple negative breast cancers compared to 14 matched normal adjacent tissue specimens (>2-fold difference, $p<0.05$ and a false discovery rate (FDR) = 5.0%). miRNAs differentially expressed in lymph node negative patients are shown in bold.

Systematic name	Fold change	p-value
hsa-let-7a	-2.36	0.00419
hsa-let-7b	-2.99	0.00011
hsa-let-7c	-3.84	0.00006
hsa-miR-100	-4.37	0.00176
hsa-miR-101	-2.61	0.01750
**hsa-miR-106b**	**2.41**	**0.03725**
hsa-miR-10a	-2.37	0.02228
**hsa-miR-10b**	**-5.64**	**0.00006**
hsa-miR-125b	-5.18	0.00010
hsa-miR-126	-2.72	0.00451
hsa-miR-126*	-2.31	0.04409
hsa-miR-127-3p	-5.42	0.00463
hsa-miR-130a	-3.32	0.01446
**hsa-miR-130b**	**2.82**	**0.00602**
hsa-miR-135b	14.14	0.00012
hsa-miR-136	-6.18	0.00011
hsa-miR-140-3p	-2.21	0.04303
**hsa-miR-141**	**2.08**	**0.04343**
hsa-miR-143	-2.99	0.01750
**hsa-miR-145**	**-6.30**	**0.00050**
**hsa-miR-183**	**5.80**	**0.00199**
hsa-miR-18b	4.67	0.04140
hsa-miR-195	-5.70	0.00006
hsa-miR-1977_v14.0	-2.28	0.00377
hsa-miR-199a-3p	-2.50	0.01750
hsa-miR-199a-5p	-2.46	0.04409
hsa-miR-199b-5p	-5.66	0.00011
hsa-miR-203	4.47	0.02087
hsa-miR-205	-4.65	0.02890
**hsa-miR-21**	**3.07**	**0.00366**
hsa-miR-210	4.56	0.00862
hsa-miR-214	-2.64	0.01360
hsa-miR-26a	-2.67	0.01428
hsa-miR-26b	-2.43	0.01681
hsa-miR-29c	-2.33	0.04343
hsa-miR-320c	-2.15	0.02461
hsa-miR-34a	-2.12	0.02087
hsa-miR-376a	-5.12	0.04809
hsa-miR-376c	-5.56	0.04809
hsa-miR-432	-2.65	0.00377
hsa-miR-451	-8.36	0.00050
hsa-miR-497	-5.11	0.00006
hsa-miR-542-3p	-3.41	0.01360
**hsa-miR-93**	**2.67**	**0.01446**
**hsa-miR-96**	**4.57**	**0.00366**
**hsa-miR-99a**	**-5.64**	**0.00011**

Supplementary Table S6: miRNAs differentially expressed in lymph node metastases. Fold change in expression of 63 miRNAs found to be significantly different in extracts from 13 lymph node metastases compared to 14 matched normal adjacent tissue specimens (>2-fold difference, $p<0.05$ and a false discovery rate (FDR) = 5.0%).

Systematic name	Fold change	p-value
hsa-let-7a	-3.33	0.00042
hsa-let-7b	-4.67	0.00007
hsa-let-7c	-6.34	0.00001
hsa-let-7e	-2.07	0.03712
hsa-let-7f	-2.26	0.02032
hsa-miR-100	-4.57	0.00331
hsa-miR-101	-3.30	0.02278
hsa-miR-10a	-4.64	0.00496
hsa-miR-10b	-8.61	0.00021
hsa-miR-1202	-3.07	0.00410
hsa-miR-1207-5p	-2.08	0.02278
hsa-miR-1225-5p	-2.52	0.00331
hsa-miR-125b	-8.41	0.00001
hsa-miR-126	-3.76	0.00451
hsa-miR-126*	-2.63	0.02725
hsa-miR-1275	-2.87	0.00235
hsa-miR-1305	-2.12	0.04847
hsa-miR-130a	-6.61	0.00430
hsa-miR-134	-2.32	0.02023
hsa-miR-135b	6.92	0.02725
hsa-miR-136	-9.58	0.00223
hsa-miR-140-5p	-2.15	0.02725
hsa-miR-143	-3.04	0.03718
hsa-miR-145	-7.86	0.00062
hsa-miR-1826	-2.61	0.01068
hsa-miR-183	5.20	0.00331
hsa-miR-188-5p	-2.38	0.01123
hsa-miR-195	-10.02	0.00029
hsa-miR-1977_v14.0	-2.91	0.00069
hsa-miR-199a-3p	-4.84	0.00451
hsa-miR-199a-5p	-4.64	0.00869
hsa-miR-199b-5p	-9.29	0.00021
hsa-miR-205	-5.18	0.01648
hsa-miR-210	2.98	0.02389
hsa-miR-214	-3.64	0.02023
hsa-miR-221	-2.96	0.02799
hsa-miR-224	4.22	0.03274
hsa-miR-23b	-3.40	0.02023
hsa-miR-26a	-3.84	0.00247
hsa-miR-26b	-3.01	0.01308
hsa-miR-29c	-2.69	0.03676
hsa-miR-30a	-2.83	0.02278
hsa-miR-30a*	-2.71	0.02799
hsa-miR-30b	-2.48	0.02799
hsa-miR-30c	-2.52	0.02309
hsa-miR-320a	-2.42	0.02356
hsa-miR-320b	-2.35	0.02023
hsa-miR-320c	-2.65	0.02023
hsa-miR-320d	-2.19	0.02452
hsa-miR-34a	-3.59	0.00825
hsa-miR-34b*	-2.60	0.02799
hsa-miR-361-5p	-2.57	0.02278
hsa-miR-424	-7.64	0.00158
hsa-miR-494	-2.57	0.00331
hsa-miR-497	-7.91	0.00029
hsa-miR-513a-5p	-3.11	0.00112
hsa-miR-513b	-3.18	0.00858
hsa-miR-572	-2.38	0.00057
hsa-miR-638	-2.31	0.00029
hsa-miR-671-5p	-2.14	0.01748
hsa-miR-886-3p	-2.79	0.02877
hsa-miR-96	3.79	0.00421
hsa-miR-99a	-10.84	0.00001

Supplementary Table S7: TAM analysis for enriched functional categories of the 27 differentially expressed miRNAs that are associated with lymph node metastases.

Function	No. of miRNAs	Fold over-representation	FDR corrected p-value	miRNAs involved
miRNAtumor suppressors	11	6.99	1.07E-05	let-7a, let-7b, let-7c, miR-125b, miR-126, miR-26b, miR-26a, miR-143, miR-195, miR-101, miR-34a
Folliculogenesis	5	16.80	1.91E-04	let-7a, let-7b, let-7c, miR-125b, miR-143
Human embryonic stem cell (hESC) regulation	13	3.60	4.14E-04	miR-125b, miR-126, miR-135b, miR-10a, miR-214, miR-199a, miR-26b, miR-26a, miR-199b, miR-143, miR-210, miR-195, miR-34a
Cell death	9	3.85	7.32E-03	let-7a, let-7b, let-7c, miR-125b, miR-497, miR-205, miR-143, miR-210, miR-34a
Angiogenesis	5	4.90	0.036	let-7b, miR-205, miR-34a, miR-126, miR-130a
Cell cycle related	8	2.85	0.0493	let-7a, let-7b, miR-125b, miR-205, miR-143, miR-210, miR-195, miR-34a
Adipocyte differentiation	5	4.36	0.0496	let-7a, let-7b, let-7c, miR-143, miR-130a

Supplementary Figure S1: Photomicrographs of a representative triple negative breast cancer case. Haemotoxylin and eosin stained sections (20x magnification) of **A)** normal adjacent tissue (NAT), invasive ductal carcinoma (IDC) and a lymph node metastasis (LN met) from a single patient diagnosed with TNBC. The black bar represents 2mm and the black square shows the magnified area in the adjacent photomicrograph. NAT- black arrow indicates stroma, white arrow indicates a terminal ductal lobular unit. LN met- black arrow indicates invasive cancer, white arrow indicates normal lymph node. **B)** Immunohistochemical detection of ER, PR and HER2, and chromosome *in situ* hybridisation (CISH) for HER2 (100x magnification).

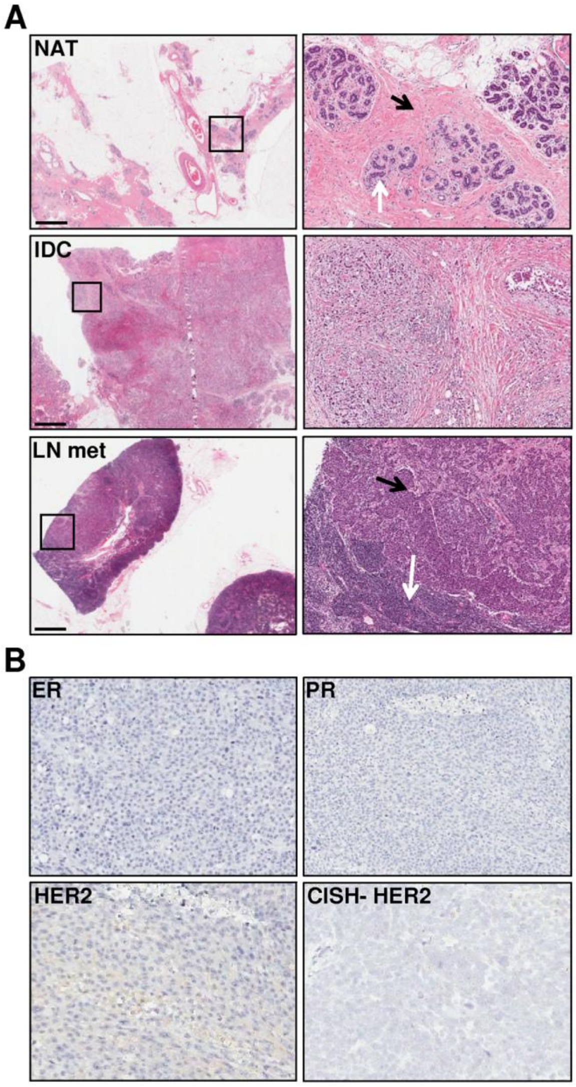

Supplementary Figure S2: Venn diagram showing the overlap between gene targets predicted by Target Scan, Pictar and miRanda.

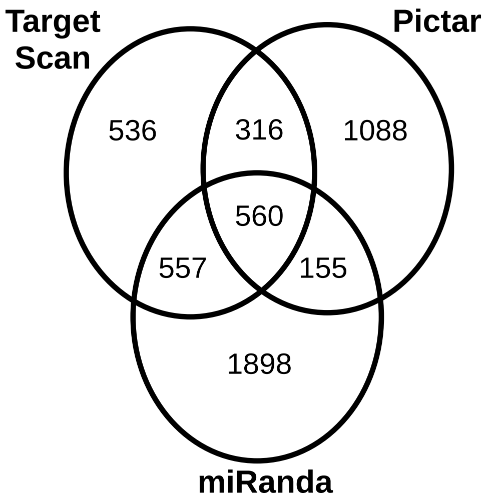

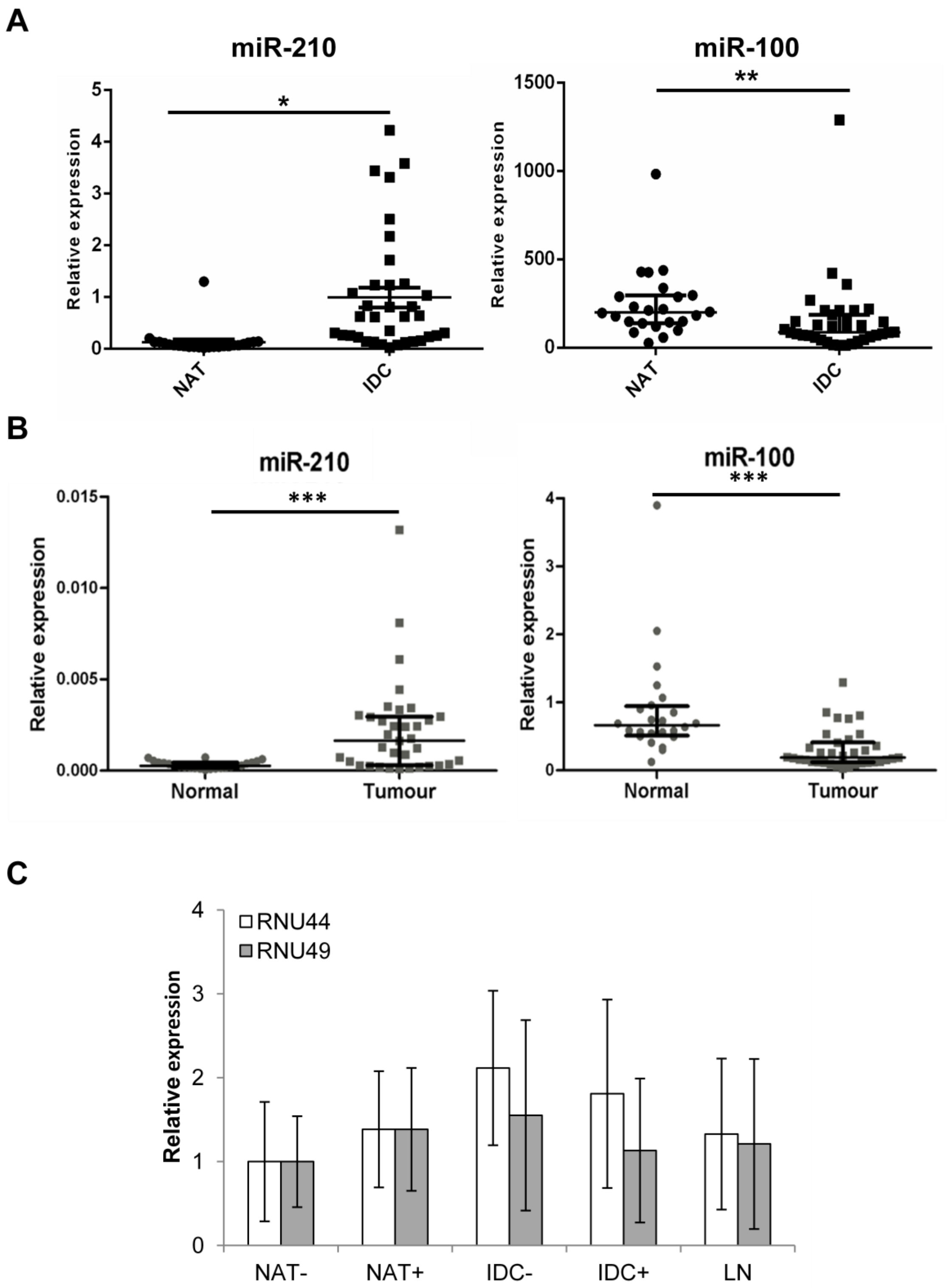

Supplementary Figure S3: Validation of RNU44 and RNU49 as normalisers. Relative quantification of miR-210 and miR-100 by real-time RT-PCR in normal (n=24) and tumour samples (n=35). Results are shown as a scatter plot of the relative normalised expression of the target miRNA to **A)** RNU 44 and **B)** RNU49 ($2^{-\Delta Ct}$). Values represent the median ± interquartile range. *p<0.0006, **p=0.0016, ***p<0.0001. **C)** The relative expression of RNU44 (white bars) and RNU 49 (grey bars) in NAT and IDC from lymph node negative tissues (-) and lymph node positive tissues (+). Values represent the mean ± SD.

Supplementary Figure S4: Correlation between real-time PCR on uniplex non-amplified cDNA and multiplexed pre-amplified cDNA. Correlation of Ct values using **A)** 5ng or **B)** 25ng total T-47D RNA. Statistical significance was determined using Spearmans' correlation. **C)** Relative quantification of individual miRNAs with (grey) or without (black) pre-amplification. Ct values for non-preamplified assays were adjusted for the median Ct gain for each assay (Supplementary Table 1) before calculating the relative expression levels. Results are shown as the mean relative normalised expression (target/RNU44) of the target miRNA ($2^{-\Delta Ct}$) ± SD.

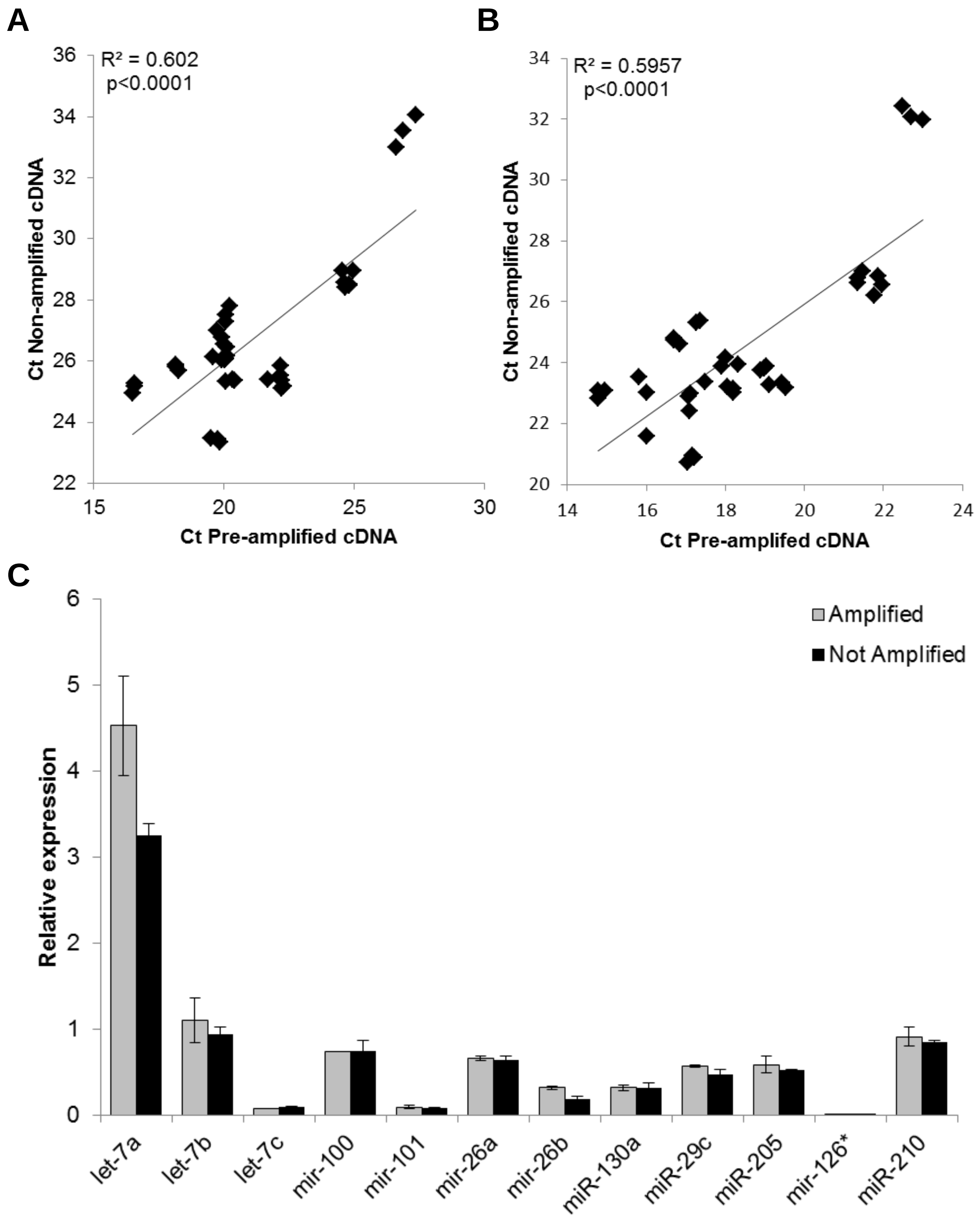

Supplement: Additional file 1 — Further characterisation of miRNAs in triple negative breast cancer. Contains Supplementary methods, Tables S1-S7 and Figures S1-S4. [file 1471-2407-14-51-S1.pdf]
